# Uncovering protein function: from classification to complexes

**DOI:** 10.1042/EBC20200108

**Published:** 2022-08-10

**Authors:** Rhiannon Morris, Katrina A. Black, Elliott J. Stollar

**Affiliations:** 1 Walter and Eliza Hall Institute, 1G Royal Parade, Parkville, 3052, VIC, Australia; 2 The University of Melbourne, School of Medicine, Dentistry and Health Sciences, Royal Parade, Parkville, 3050, VIC, Australia; 3 School of Life Sciences, University of Liverpool, U.K.

**Keywords:** biochemical techniques and resources, post translational modification, protein binding

## Abstract

Almost all interactions and reactions that occur in living organisms involve proteins. The various biological roles of proteins include, but are not limited to, signal transduction, gene transcription, cell death, immune function, structural support, and catalysis of all the chemical reactions that enable organisms to survive. The varied roles of proteins have led to them being dubbed ‘the workhorses of all living organisms’. This article discusses the functions of proteins and how protein function is studied in a laboratory setting. In this article, we begin by examining the functions of protein domains, followed by a discussion of some of the major classes of proteins based on their function. We consider protein binding in detail, which is central to protein function. We then examine how protein function can be altered through various mechanisms including post-translational modification, and changes to environment, oligomerisation and mutations. Finally, we consider a handful of the techniques employed in the laboratory to understand and measure the function of proteins.

## Introduction

Proteins are one of the major classes of macromolecules and are essential for a diverse range of biological functions. The unique amino acid sequence of proteins determines their global 3D structure, which in turn defines their cellular function. Each protein has a distinctive structure and size. To enable a full understanding of the role of proteins within cells, it is necessary to explore protein structure alongside function. We therefore recommend also reading the ‘uncovering protein structure’ review article in this series for an overview of the structural concepts governing protein function, although some structural concepts will also be discussed here in less detail. In this review, we will discuss the functions of protein and their constituent domains, how they bind to their targets, and how post-translational modification, environment, and localisation can affect protein function. We will then consider how mutations and modifications to proteins can have large effects on their function as well as broader biological processes. Importantly, a large portion of this review will focus on the interactions of proteins with other molecules, including other proteins, and how these interactions are studied in the laboratory to further understand protein function.

**Figure 1:**
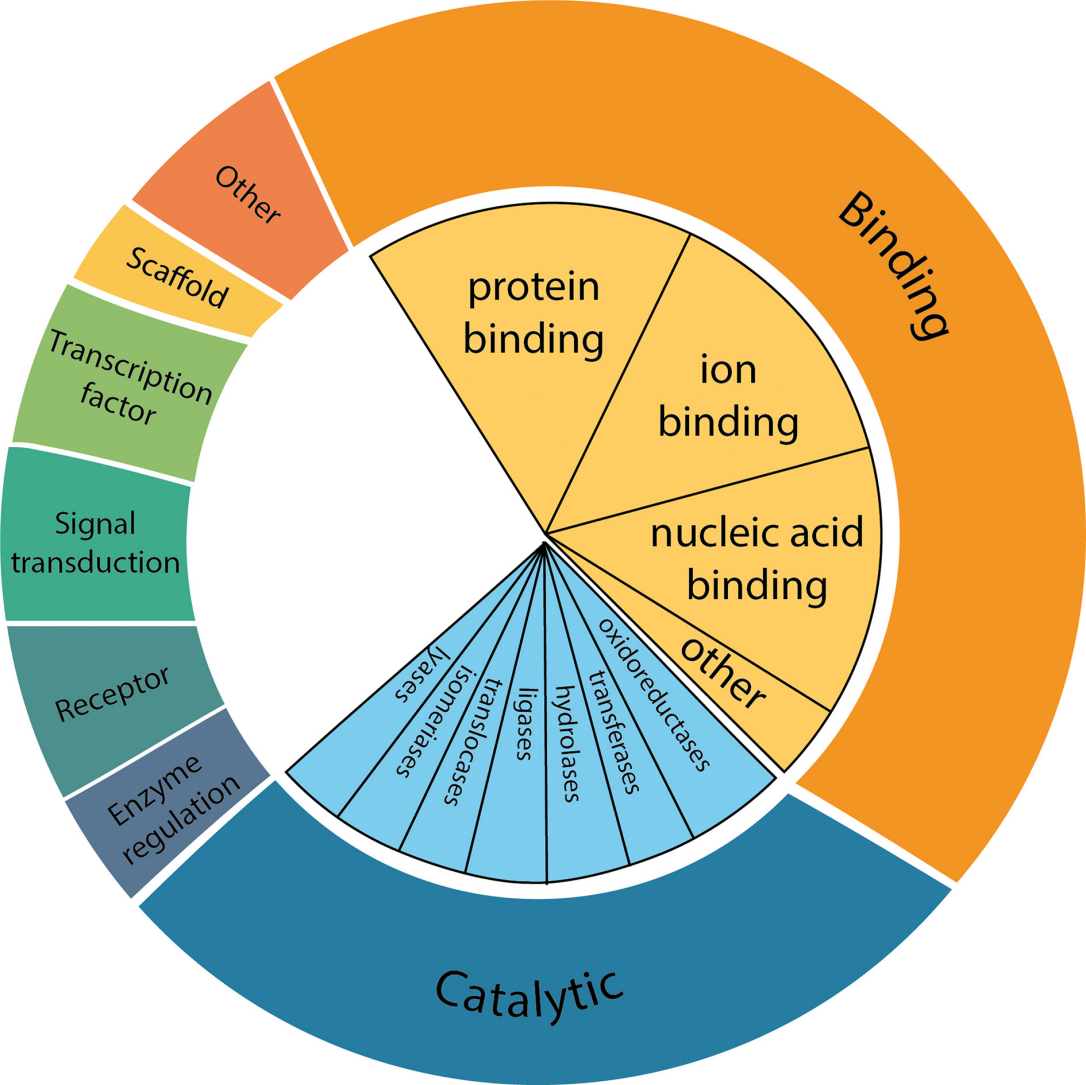
Classification of human proteins by function Proteins tend to be classified based on the biological function they perform. These groups can then be further divided into subsections. Enzymes can be further divided into groups based on the reactions they catalyse, and binding proteins based on their ligands, or binding partners. The subcategories for enzymes are from the internationally agreed enzyme classification system from the Enzyme Commission (EC).

Before discussing protein function further, it is important to define what exactly is meant here by ‘function’. From an evolutionary perspective, the function of any protein is to enhance the fitness of the host organism. However, in this article we discuss protein function in terms of its specific biological role in the cell. For instance, a protein may provide structural support, move molecules in or out of a cell or cellular compartment, enable communication within an organism or catalyse metabolic reactions. These are all examples of protein function. Given the vast array of protein functions, it should be stated at the outset that the examples discussed in this review are by no means exhaustive. It is instead our hope that the examples given here will provide insight into the large functional repertoire of proteins. A useful resource for appreciating the distribution of proteins by function is the Gene Ontology database (http://geneontology.org/) that associates each protein in a given organism with a molecular function, biological process and cellular compartment. As shown in Figure 1, most human proteins fall into two broad categories: enzymes and proteins that bind other biological molecules. However, there are also several other categories, and in some cases proteins may fit into multiple categories. Additionally, in some ways, all proteins interact with other molecules as they exist within cells, a biological environment.

## Classifying protein function

**Figure 2:**
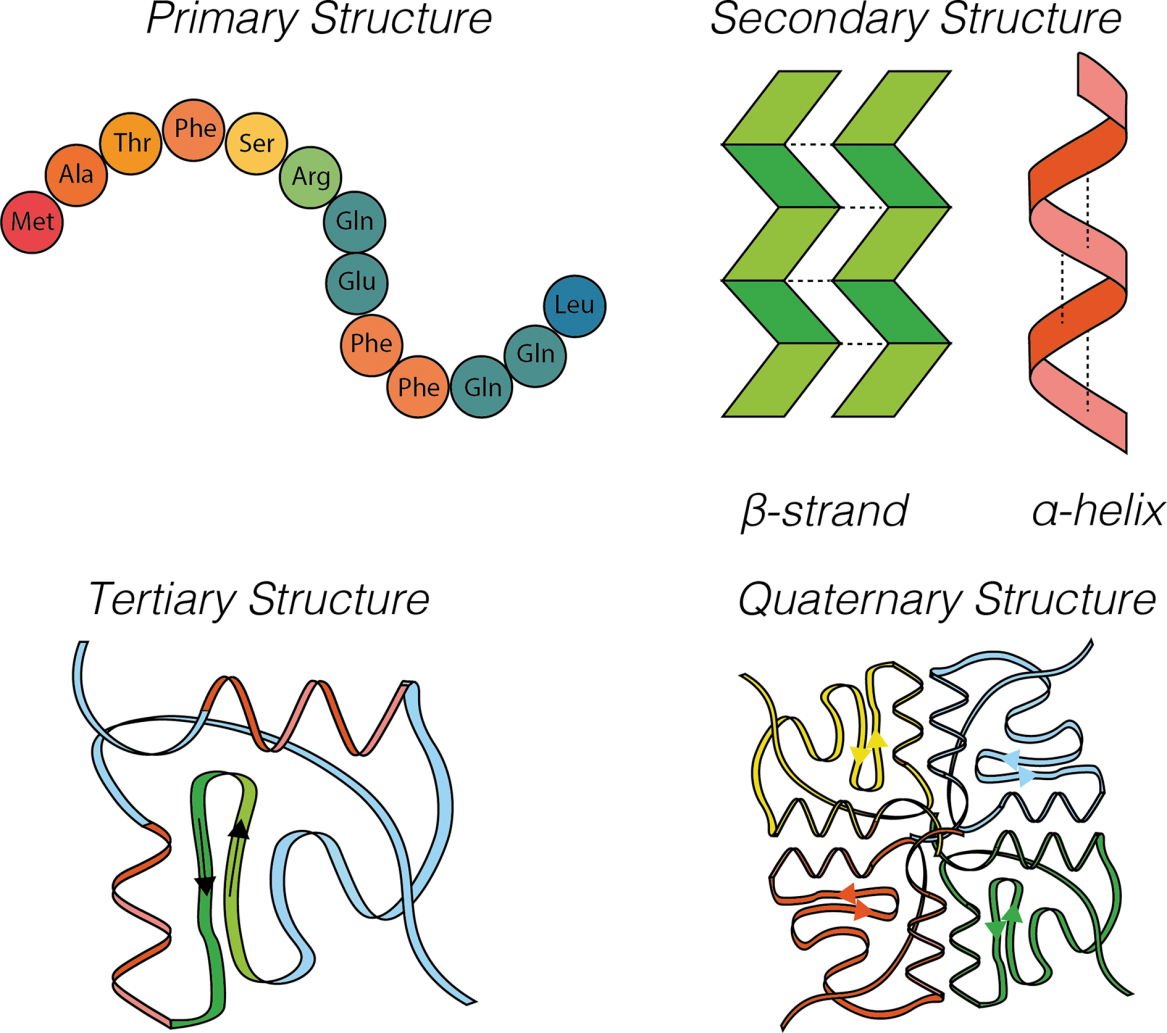
Primary, secondary, tertiary, and quaternary protein structure Primary structure is the sequence of a chain of amino acids. Secondary structure is due to hydrogen bonding of the peptide backbone, allowing the peptide to fold into a repeating structure such as α-helices or β-sheets. Tertiary structure is the 3D fold of a protein that occurs due to side chain interactions and core packing. Quaternary structure occurs when multiple chains, or subunits, interact to form a functional complex. Here, each polypeptide chain is depicted in a unique colour in a quaternary complex.

Before considering the classification of protein function, it is useful to briefly review structural classification of proteins. The overall 3D structure of a protein, its tertiary structure, is determined the by arrangement of the primary and secondary structural elements (Figure 2). The amino acid sequence that makes up the polypeptide chain of a protein is termed the ‘primary structure’. The ‘secondary structure’ is the formation of regular, recurring structural elements (α-helices and β-sheets) by interactions between the atoms of the peptide backbone, that is, the peptide chain excluding the variable sidechains. Both β-sheets and α-helices are maintained through hydrogen bonding between carbonyl oxygen atoms and amino hydrogen atoms of the backbone. The overall structure of the entire protein is termed the ‘tertiary structure’ and is primarily mediated by interactions between amino acid side chains. These interactions include hydrogen bonds, ionic interactions, van der Waals interactions, disulfide bonds and hydrophobic interactions that induce protein folding. Finally, the ‘quaternary structure’ of proteins is a structural arrangement that occurs through association of more than one polypeptide chain to form a single functional unit. In this case, the individual chains in the complex are referred to as subunits. Most proteins consist of smaller units called ‘domains’. Classification of domains is typically based on the aforementioned structural features (see ‘Uncovering protein structure’ in this series for more detail). However, protein domains can also be classified based on their function. As such, we consider specific protein domains as isolated units in more detail in the next section.

### Protein domains and their functions

Domains are modular components of proteins that can fold independently of the rest of the polypeptide chain. Generally, one type of domain will have similar properties in all proteins, regardless of the relatedness of the protein’s overall function. Some proteins comprise only a single domain that performs a highly specialised function, while many others are made up of several domains that each serve a distinct purpose that together, allow the protein to perform a sophisticated function. In some cases, proteins within a family will contain the same domains, giving them the same overall function. However, small differences in the primary sequence of the domains between family members will allow each family member to be involved in a different biological process.

**Figure 3:**
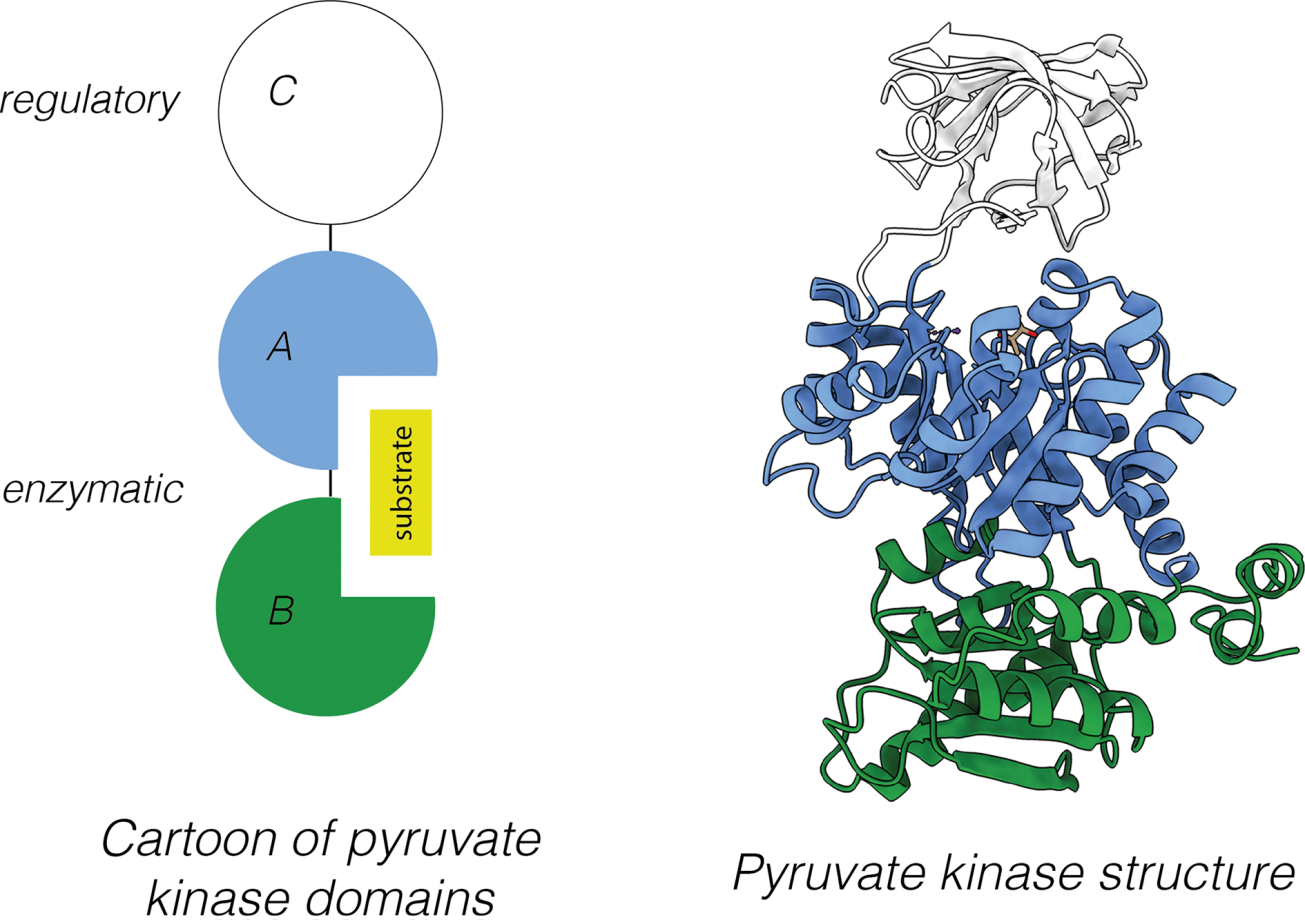
The three distinct domains of pyruvate kinase Pyruvate kinase is arranged into three domains, shown here in blue (domain A), green (domain B), and white (domain C). The domains are illustrated through a schematic diagram (left) or with structural models (right). Domain C is the regulatory domain and binding of fructose-1,6-bisphosphate to domain C activates the catalytic activity of pyruvate kinase due to a conformational change. Together domains A and B form an enzymatic active site and perform a catalytic function. Despite the different functions of the individual domains of pyruvate kinase (regulatory and catalytic), the overall function of the protein is typically considered to be enzymatic; PDB ID: 1PKN.

Protein domains can be characterised by sequence, structure, or function and are often physically discrete within a given protein. Consider pyruvate kinase, the enzyme that catalyses the last step of glycolysis (transfer of a phosphate from phosphoenol pyruvate to ADP producing pyruvate and ATP). Pyruvate kinase has 3 distinct domains, designated A, B, and C (Figure 3). Domain A is the largest and together with domain B, forms the active site of this enzyme. Domain C contains an allosteric regulatory site ∼40 Å away from the active site. Binding of fructose-1,6-bisphosphate to domain C changes the conformation of the whole protein and activates pyruvate kinase activity. This concept of allosteric regulation is discussed later in this review.

### Domains with catalytic functions

As illustrated by pyruvate kinase, many domains have an enzymatic function i.e., they catalyse a specific chemical reaction within their active (catalytic) site. These catalytic domains contain specific amino acids motifs within their active site that influence their catalytic properties including substrate affinity and turnover rate. Typically, catalytic domains are found in multi-domain proteins, as seen for pyruvate kinase. The other domains may be involved in other functions such as regulation of the enzymatic domain or interacting with other biological molecules.

### Domains involved in binding

As previously highlighted, a large proportion of protein function involves binding to other proteins or biomolecules. In biochemistry, we often refer to the targets of a protein as their binding partners. Given the central role binding plays in protein function, many proteins contain domains that are exclusively involved in binding. Here, we briefly discuss some examples of domains involved in binding. The later protein binding section of this review also contains further information about how association of molecules can further influence their function.

#### Domains involved in PPIs

Domains that are involved in PPIs are important for bringing multiple proteins together in a highly specific manner. Many of these domains are necessary for the formation of homomeric and heteromeric protein complexes. Examples of these kinds of domains include SH3 domains that bind to proteins that contain proline rich motifs; sterile α motif (SAM) domains that interact with other SAM domains to form homo- or hetero-oligomers; and LIM domains, which comprise contiguous zinc fingers that interact with proteins containing PDZ domains or other LIM domains.

#### Domains that bind modified residues

**Figure 4:**
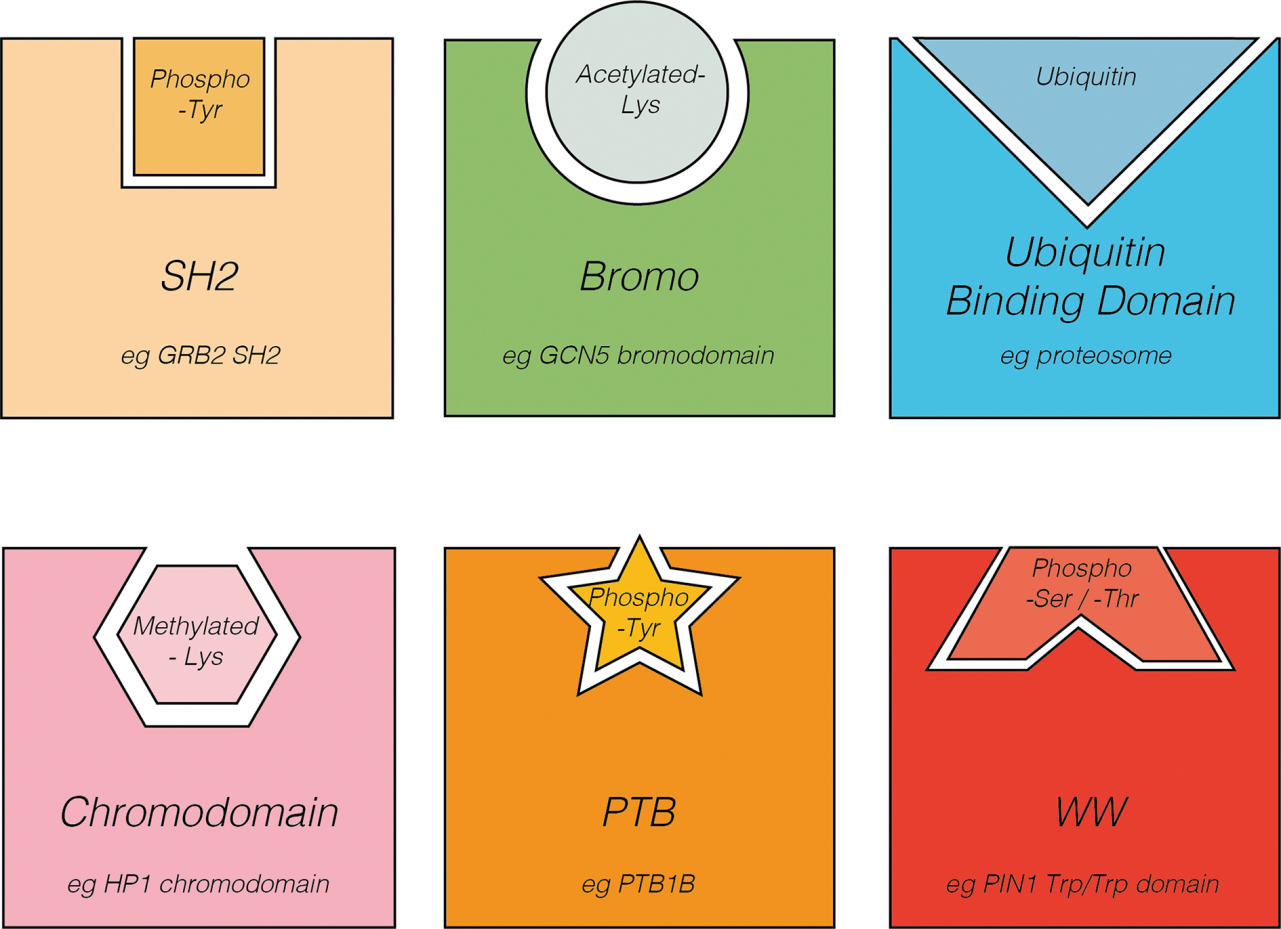
Protein domains that bind modified residues Schematic diagram of select examples of domains that bind modified residues and examples of proteins that contain this type of domain. Binding depends on a match in both shape and chemical nature between the domain and the modified residue, which is represented by shape and colour in this figure. SH2 and PTB domains bind phosphotyrosine residues. WW domains mediate binding to phosphoserine and phosphothreonine residues, while chromodomains bind methylated lysine residues. UBA domains bind ubiquitin molecules and bromodomains allow interactions with acetylated lysine residues.

Reversible, covalent modifications of residues within domains are essential signals that occur in response to the ever-changing environment within and outside cells. Some examples of modification include phosphorylation, methylation, acetylation, and lipidation (see the post-translational modification section below for more detailed examples of modifications). To propagate these signals, it is important for proteins to be able to bind these modified amino acid residues. Domains that bind to modified residues therefore allow rapid and complex PPIs in response to various stimuli, particularly in signal transduction pathways where specific amino acids within a protein are rapidly modified. Domains that bind modified residues are typically components of multi-domain proteins and serve to form PPIs. Some examples of domains that interact with modified residues are listed in Figure 4.

**Figure 5:**
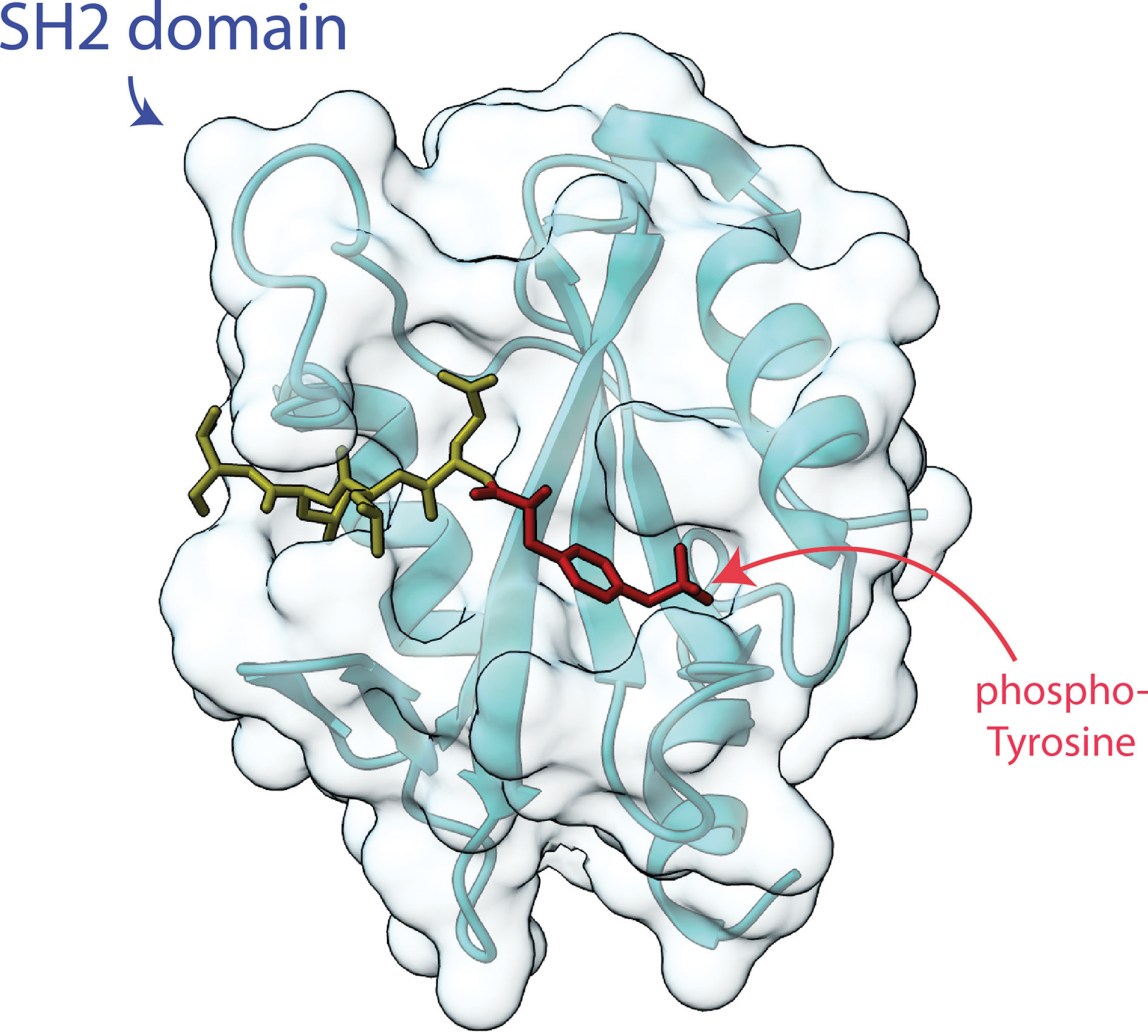
LNK (SH2B3) SH2 domain with phosphopeptide bound Cartoon representation of the backbone of an SH2 domain with the secondary structural features indicated beneath a surface representation. LNK SH2 domain structure with the JAK2 pY813 peptide shown; PDB ID: 7R8W.

An example of a domain that binds a modified residue is the Src Homology 2 (SH2) domain. SH2 domains are phosphotyrosine binding domains that were first identified in the viral protein Src and are thus named Src Homology 2 domains. SH2 domains bind linear peptide motifs containing phosphorylated tyrosine residues and the specificity of the interaction is derived by the residues flanking the target phosphotyrosine. Structurally, SH2 domains tend to be highly conserved, comprising three central β-strands flanked by two α-helices (Figure 5). Approximately 100 proteins containing SH2 domains have been identified in humans, thus, developing specificity is crucial to ensure that the correct binding interaction occurs (there is more discussion of this in the protein binding section of this review). SH2 domains are typically found in proteins involved in phosphotyrosine dependent signal transduction and serve as modulators of PPIs.

#### Domains that interact with other biological molecules

**Figure 6:**
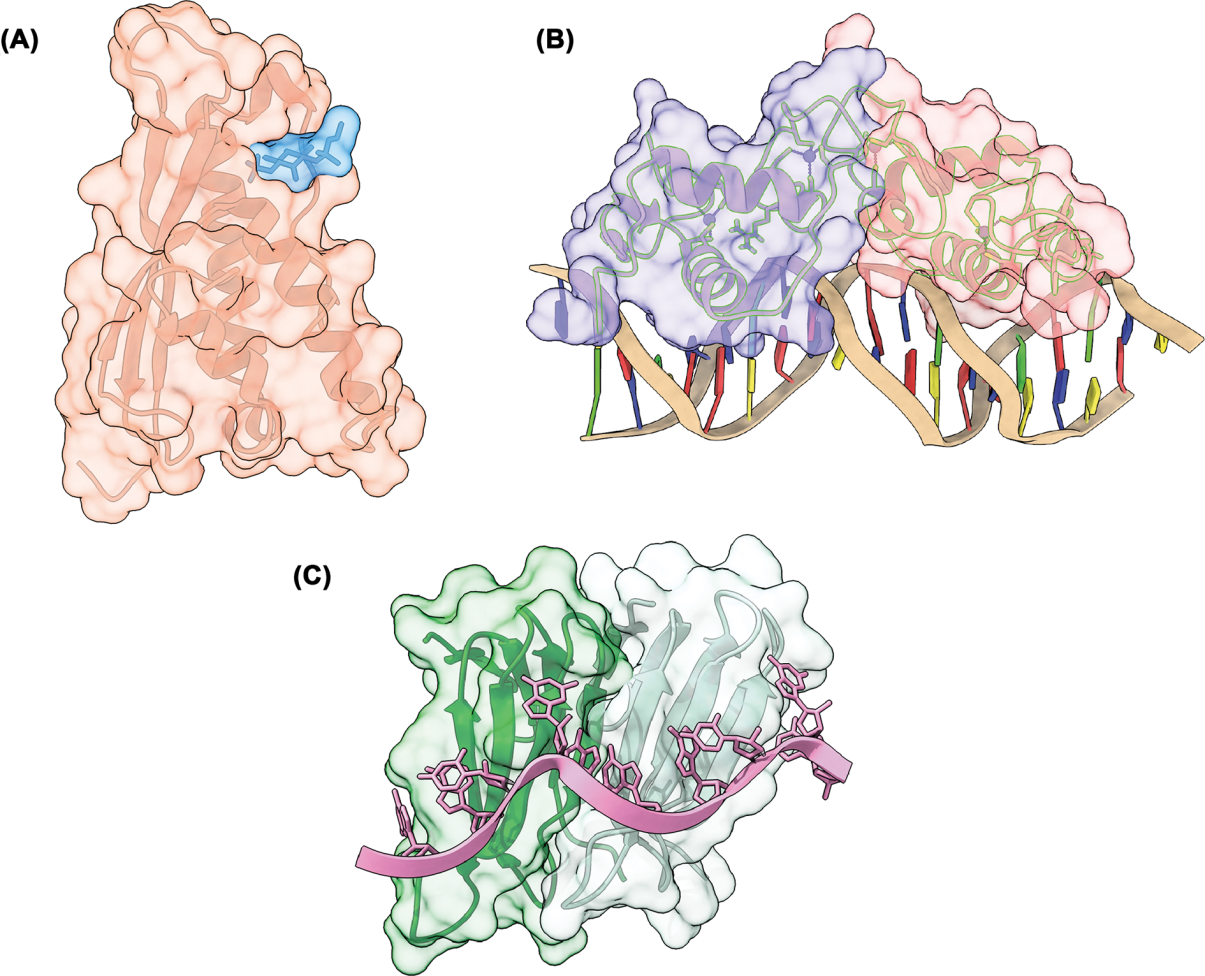
Protein domains that bind other biological molecules Select examples of protein domains binding other macromolecules. (**A**) The PX domain of a yeast sorting nexin (orange) binding to the headgroup of a lipid, PtdIns3P (blue), PDB ID: 1OCU. (**B**) The glucocorticoid receptor, a homodimer, (purple, red) bound to a DNA molecule (tan helix), PDB ID: 1R4R. (**C**) The Trp RNA-binding attenuation protein (TRAP) (green) bound to an RNA molecule (pink helix), PDB ID: 1C9S.

In addition to mediating PPIs, which we abbreviate to PPIs, the function of a domain may depend on interactions with other macromolecules, such as nucleic acids or lipids. Interactions between proteins and these other molecules are important for a myriad of biological processes including catalysis, gene transcription and translation. Additionally, such interactions are key for sequestering or localising proteins to certain cellular compartments. For example, some protein–lipid interactions embed proteins in specific membranes within the cell. Some examples of domains that form interactions with other molecules are shown in Figure 6. The later section on protein binding goes into further detail on how protein binding to lipids and nucleic acids can impact their function.

### Domains with regulatory functions

Multi-domain proteins, particularly those with enzymatic function often contain regulatory domains. The role of these domains is to specifically regulate catalysis. An example of a regulatory domain was exemplified in Figure 2, where domain C of pyruvate kinase regulates catalytic activity by sequestering the B domain, which is critical for active site formation. Some regulatory domains allow conformational changes in the protein as seen for pyruvate kinase, maintaining the protein in an autoinhibited state.

### The diverse functions of proteins

**Figure 7:**
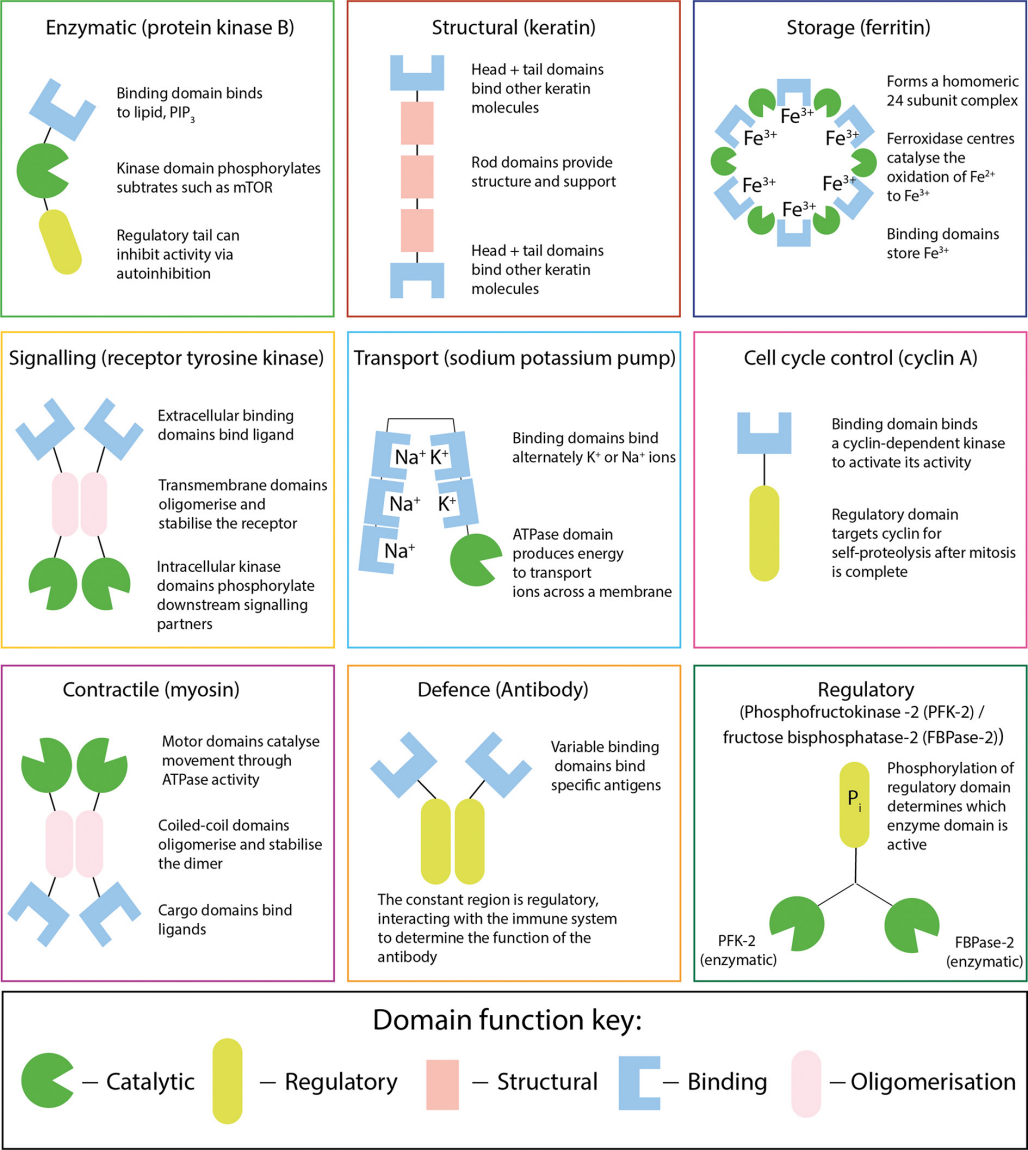
Examples of the diverse functions of proteins and the domains they comprise Many proteins are made up of multiple functional domains that come together to give rise to the overall function of the protein. Shown above is a schematic representation of how catalytic, regulatory, binding, structural and oligomerisation domains can come together to give rise to different functions in different proteins. This figure aims to show that different functional domains work together to control different elements of a protein’s function, rather than to accurately depict the protein’s structure.

Full-length proteins can be made up of one or multiple domains that give rise to the overall function of a protein (Figure 7). In this section, we will provide some examples of how proteins can be placed into different functional groups, and where appropriate, we will discuss how the different functions of the individual domains give rise to the overall protein function. However, it should be noted that many of these proteins can fall into more than one of the groups listed, for example, many signalling proteins, such as protein kinases, are also enzymes. Here, we have placed each into what we see as the primary function of the protein for ease of explanation.

### Enzymes

Cells rely on enzymes to catalyse all reactions that are essential for life. Enzymes typically bind molecules (called substrates) and facilitate a chemical reaction. We suggest referring to the enzymes review in this series to learn more about the main categories of enzymes and the reactions they catalyse. Enzymes work by lowering the activation energy of a reaction, allowing the reaction rate to be increased. In many cases, an enzyme requires cofactors or coenzymes to function. Coenzymes are organic molecules mostly derived from vitamins whereas cofactors are inorganic species, such as the metal ion magnesium. Humans and some other animals cannot synthesise all coenzymes necessary for life and they must be ingested through a balanced diet. The term ‘apoenzyme’ is used to denote an enzyme that is not bound to a cofactor or coenzyme, and likewise, ‘holoenzyme’ is used to refer to the bound state.

As an example, the enzyme methylmalonyl-CoA mutase requires vitamin B12 for its isomerisation reaction, and branched-chain 2-oxo acid dehydrogenase is inactive without its cofactor vitamin B1.

Table 1 highlights examples of multidomain proteins and their catalytic domains. It should also be noted that some enzymes only contain one functional domain, an example of this being lysozyme. Lysozyme is also an example of a protein that functions as an enzyme, but whose cellular role is defence.

**Table 1:** Examples of enzymes and their catalytic domains

Enzyme	All domains	Catalytic domain	Function
**AKT**	Plekstrin homology, Kinase	Kinase	Catalyses the addition of phosphate groups onto tyrosine residues in target proteins
**SHP2**	SH2 and phosphatase	Phosphatase	Catalyses dephosphorylation of phosphotyrosine residues in target proteins
**CESA3**	Zinc finger, transmembrane and glycosyltransferase	Glycosyltransferase	Catalyses the synthesis of cellulose

### Structural proteins

Some of the most abundant proteins in nature have structural functions within cells and organisms. These proteins are essential for both the formation and maintenance of cellular structure, forming extensive networks particularly in eukaryotic cells. In humans, extracellular collagen is the principal protein of connective tissues and accounts for approximately 25% of total protein mass, highlighting the abundance and importance of these structural proteins. In addition to maintaining cellular structure, a number of structural proteins are also essential for the distribution of organelles throughout the cytoplasm and play an important role in alignment of the chromosomes along the cell centromere prior to cytokinesis during cell division. Table 2 provides some examples of structural proteins and their specific functions.

**Table 2:** Examples of structural proteins and their functions

Protein	Function
**Actinin**	Attachment of actin filaments to skeletal muscle
**Cadherin**	Formation of cell–cell junctions
**Elastin**	Cell structure, elasticity and flexibility
**Fibronectin**	Cell adhesion, growth, migration, and differentiation
**Keratin**	Structural protein in hair, skin, and nails
**Tubulin**	Cell structure, functions in cell division

### Storage proteins

Storage proteins are important for sequestering metal ions and as a source of amino acids. Ferritin is an example protein which is produced by almost all living organisms and is important for storing iron (Figure 7). Ferritin assembles its 24-subunits into a nanocage that interacts with iron, keeping it in a non-toxic and readily available form. Proteins that function as a storage of amino acids are essential for animal embryonic development and in germinating seedlings for plants. Ovalbumin is the main protein in egg whites and functions as a storage protein. Ovalbumin contains 385 amino acids that can be released and used to build proteins during embryonic development. In plants, storage proteins accumulate in vegetive and reproductive tissues and serve as a reservoir for building materials during stages of development. Plant storage proteins can be either seed storage proteins, or vegetative storage proteins. Seed storage proteins, as their name suggests, accumulate in plant seeds and the amino acids are used as a nutritional source during development. Vegetative storage proteins, on the other hand, accumulate in leaves, stems and in some plants in tubers. An example of a plant storage protein is gluten. Gluten is a actually a mixture of many related proteins that are found in various wheat grains such as oats, barley and rye and act as an amino acid store for the plant.

### Signalling proteins

Signalling proteins are essential for cell function as they allow cells to communicate with one another and respond to cues from their environment. Signalling proteins can be found in all compartments of the cell, including the plasma membrane, the cytoplasm, the nucleus, and the mitochondria. Receptors detect signals and are often found in the plasma membrane, as many signals cannot cross the membrane. However, some receptors, like those for the estrogen hormone are localised in the cytosol, as these hormones can cross the plasma membrane. Binding of a ligand to the extracellular portion of membrane receptors leads to a change inside the cell that relays the signal from outside. This process is termed signal transduction and allows cells to communicate with one another and respond to cues from their environment in a controlled manner. Signalling proteins often contain domains that bind modified residues as discussed in the first section of this review. An example of this is the signal transducer and activator of transcription (STAT) family of proteins. The STAT proteins contain SH2 domains that bind to phosphorylated residues on receptors inside the cell, and upon binding, they too become phosphorylated at tyrosine residues, which leads to a conformational change that ultimately allows them to translocate into the nucleus, bind DNA and up-regulate DNA transcription.

### Transport proteins

All cells must transport molecules between various cellular compartments, across the plasma membrane, and in the case of multicellular organisms, between cells. Ions such as sodium and potassium, proteins such as neurotransmitters and sugars such as glucose all need to be transported in some way. Channels, pumps, and pores transport specific molecules across membranes. Molecules can also be transported within cells or throughout the body by binding to transport proteins, for example O_2_ binding to haemoglobin. Some examples of transport proteins are detailed in Table 3. For a detailed description of types of transport across membranes (i.e., facilitated, or active transport) see the Biological Membranes Review in this series.

**Table 3:** Examples of transport proteins and their functions

Protein	Function
**Dynein**	A microtubule motor protein which transports a variety of intracellular cargo by hydrolysing ATP to power its movement along microtubule tracks
**Sodium–potassium pump**	An enzyme found in the membrane of animal cells that uses ATP to pump sodium and potassium across membranes against their concentration gradients (Figure 6)
**GLUT4**	Insulin-regulated glucose transporter
**Transthyretin**	Transports the hormone, thyroxin and vitamin A (retinol) around the body
**ATP/ADP transporter**	A mitochondrial transport protein within the inner mitochondrial membrane that transports ADP and ATP as the name suggests. ADP is imported from the cytosol and ATP is exported from the mitochondria

### Regulatory proteins

**Figure 8:**
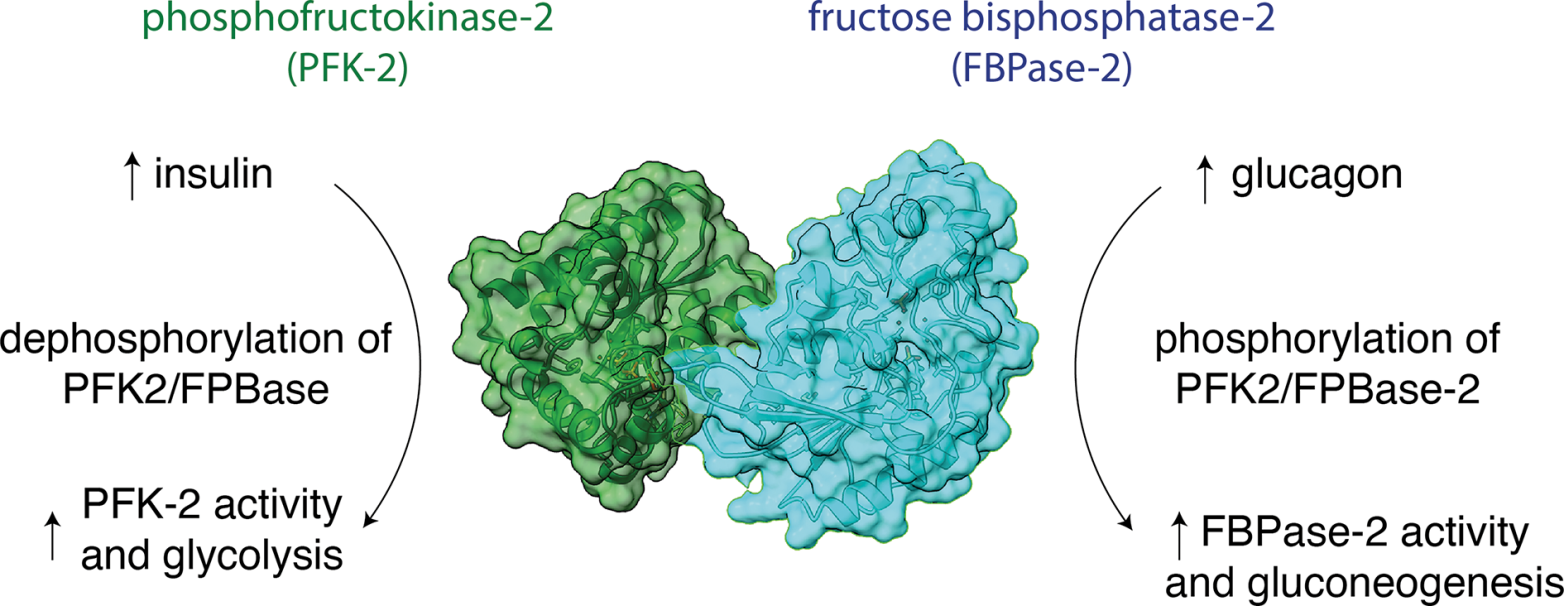
Regulation of metabolism by phosphofructokinase-2/fructosebisphosphatase-2 Glycolysis and gluconeogenesis are two key metabolic processes that regulate energy levels in the body through cellular respiration. Glycolysis is the process whereby glucose is broken down and is the first step in respiration in eukaryotes, whereas gluconeogenesis allows glucose to be made from simpler precursors such as lactate or pyruvate. Phosphofructokinase-2/fructosebisphosphatase-2 is a bifunctional enzyme that is a key regulator of glycolysis and gluconeogenesis. Both phosphofructokinase-2 (green, left) and fructosebisphosphatase-2 (blue, right) are part of the same 55 kDa polypeptide chain that contains an N-terminal regulatory domain, a kinase domain and a phosphatase domain. Phosphofructokinase-2/fructosebisphosphatase-2 catalyses the formation and degradation of the key allosteric regulator fructose-2,6-bisphosphate, which acts as a mechanism for switching between glycolysis and gluconeogenesis. In brief, when blood sugar is high (depicted on the left of the figure), insulin is produced. A downstream effect of insulin signalling results in dephosphorylation of the PFK-2/FBPase-2 complex and increased PFK-2 kinase activity. This results in increased fructose-2,6-bisphosphate levels and increased glycolysis. Alternately, when blood glucose is low (depicted on the right of the figure), the hormone glucagon is produced. This results in phosphorylation of the complex and increased FBPase-2 phosphatase activity. This decreases fructose-2,6-bisphosphate levels, slowing glycolysis and increasing gluconeogenesis.

Almost all proteins require regulation to ensure they perform their function at the right time and place. We have already seen how domains can mediate PPIs and how protein modifications can allow switching between functional states. Regulation of gene expression is an excellent example how regulatory proteins control a central cellular function. Here, transcription factors and other DNA-binding proteins interact with DNA to control when and to what extent a gene is transcribed and/or which epigenetic signals are made on the DNA. Regulatory proteins can also regulate the flux of a signalling or metabolic pathway, often significantly altering biological outcomes. Other regulatory proteins include molecules such as cyclins, which control progress through the cell cycle. Cyclins activate cyclin dependent kinases which phosphorylate the proteins that drive the cell cycle, such as the DNA replication machinery. Many proteins’ primary function is to regulate other pathways or functions in the cell, thus maintaining homeostasis. Below we use the example of phosphofrutokinase-2/fructose bisphosphatase-2 to illustrate the role of a regulatory protein in maintaining homeostasis in a biological system (Figure 8).

### Defence proteins

Defence proteins are found in a wide variety of organisms, and despite mechanistic diversity, all act to defend an organism from foreign attack. The venoms of snakes and spiders contain a cocktail of defence proteins that disable predators, enabling escape. Bacteria such as *Streptococcus pyogenes* contain an elaborate molecular defence system known as the Clustered Regularly Interspaced Short Palindromic Repeats (CRISPR) system. CRISPR relies on the RNA-guided endonuclease Cas9 to modify the DNA of invading viruses and plasmids and has become a commonly used gene editing tool in modern molecular biology. Antibodies, or immunoglobulins, are defence proteins produced by B-cells that circulate in the blood. They form an essential component of the adaptive immune system by binding to foreign molecules and are the basis for vaccination. Antibodies are also a great example of how the overall function of a protein differs from the function of its constituent parts. Antibodies contain regions that specifically recognise antigens (foreign substance) through binding, and regions that regulate the response to antigen binding. So, while the different domains that comprise the antibody function by binding or regulation, the overall function of the antibody is defence as part of the immune system.

### Summary

This section presented some examples of the range of functional domains in proteins and described the categorisation of proteins based on their function. We hope this section has provided the foundation for understanding the following sections, which will focus on the role of binding in protein function, factors that influence protein function and how we measure protein function in the laboratory.

## Binding is central to protein function

As we have seen in prior sections, most proteins can bind reversibly through non-covalent interactions to their binding partners. These binding partners may be other proteins, small ligands, nucleic acids, lipids, or sugars that occur inside or outside of the cell. It is these interactions that bring about the molecular processes essential for life. They allow chemical reactions to occur, large complex machineries to form and signalling within and between cells. Binding is also crucial for the regulation of almost all of these processes which are in constant flux during life. Below we examine protein binding in more detail, starting with the functional consequences of binding.

### Functional consequences of protein binding

The physical binding of a protein to its binding partner can have direct functional consequences by modifying the structure or accessibility to binding or catalytic sites. Conformational changes that occur in a protein at a distant site due to binding of another ‘regulatory’ molecule are an example of allostery (where binding at one site transmits an effect to a more distal site). Here, the allosteric binding event may cause a change in the configuration of the protein’s active site and thus ability to catalyse a reaction (as seen for pyruvate kinase) or a change in one of its binding surfaces and ability to bind another binding partner. Allosteric binding is a common way to regulate protein function. A different functional consequence of binding can be to physically change the subcellular location of the protein by virtue of the location of its binding partner, i.e. binding to lipids can bring a cytosolic protein to the cellular membrane. Finally, a post-translational modification of a protein also facilitates a change in subcellular location. This is common for transcription factors which may be modified in the cytoplasm, which allows for an interaction with a factor that facilitates nuclear import, where they then carry out their function as discussed above for the STAT proteins.

### Protein binding surface

In general, protein interactions occur using binding surfaces that are between 1200 and 5000 Å^2^ and involve approximately 70% direct and 30% water mediated contacts. For PPIs, the interacting proteins have good structural and chemical complementarity that involve hydrophobic, polar, or charged interactions. Usually, long-range electrostatic interactions are most important for acting at a distance to enable the initial binding events during association and hydrogen-bonds are most important for the final short-range binding events and need to be first broken during dissociation. A PPI can occur when a protein domain binds to another domain over a broad interface or when it binds to a short disordered linear peptide motif, which may bury less surface area (such as for SH2 domains, as discussed earlier). Disordered peptide interactions are frequently used as they can easily and quickly form the required functional interaction since they have fewer 3D structural requirements compared to a folded domain. As we have seen, there are many protein binding domains that specialise in recognising short, disordered peptides in this manner and usually involve large changes in peptide conformation. The exact nature of the interacting protein surfaces and associated required conformational changes will determine the binding thermodynamics and kinetics which is considered next.

### Protein binding thermodynamics and kinetics

Like protein folding, protein binding obeys the laws of thermodynamics and kinetics, enabling comparison and categorisation of different protein interactions. For a description of thermodynamics basics, it would be useful to read the protein folding section in the uncovering protein structure review of this series. The strength of the interaction between protein and its target is described by the size of the dissociation constant (*K*_d_ with molar units). The smaller the *K*_d_ value (i.e. picomolar versus millimolar), the tighter the binding. The dissociation constant is directly related the thermodynamic quantity, the standard free energy of binding (Δ*G*^o^). When an additional hydrogen bond is added to an interaction, this results in a small change in Δ*G*^o^ but a larger change in *K*_d_. Protein interaction strength spans a wide spectrum from weak to strong and this range of binding affinities mediate a range of functions. To understand the free energy of binding in more detail, similar to any change in free energy, Δ*G*^o^ can be calculated in terms of the change in entropy and change in enthalpy. The change in binding enthalpy reflects the making and breaking of bonds during binding. Many binding events are exothermic as many new bonds are formed, although enthalpy changes are hard to predict, especially when conformation changes are required for binding. The change in binding entropy reflects the change in disorder of the molecules involved with many binding events being entropically favourable even though two free components combine into one component which is more ‘ordered’. However, it is the entropy of the entire system to consider that usually includes dynamic water molecules being released at the interface of a complex. As such, it is the balance between entropy and enthalpy changes that determines the overall affinity. In general, more extensive binding surfaces have greater opportunities for amino acid interactions between the two proteins and will therefore result in stronger binding. However, the binding thermodynamics is not the only consideration as interactions rarely reach equilibrium in the cell and we need to also consider binding kinetics. The kinetics tells us how fast the protein and target bind (associate) and how slow the protein–target complex fall apart (dissociates). Related to this is the time it takes for half of the protein–target complex to dissociate, called the half-life of the complex and is calculated as 0.693 divided by the dissociation rate constant. Thermodynamics and kinetics are linked as reflected in the formula where *K*_d_ is the ratio of the dissociation and the association rate constants, which we explore again later when we consider the design of drugs to modify protein function. With these key parameters in mind, we next define the two major PPI categories; transient and obligatory PPIs.

### Transient PPIs

Transient interactions are reversable, usually short-lived and may be weak or strong. For example, some weak binding transient interactions include reversible cell-to-cell contacts or transient signalling complexes and only exist together with half-lives of milliseconds and *K_d_* values in the milli or micromolar range. Other transient interactions are extremely strong (e.g., hormone–receptor interactions or protease–inhibitor interactions) with much longer half-lives of up to days and *K_d_* values in the sub picomolar range. Regardless of their affinity, the examples discussed are considered transient PPIs as the interacting proteins are not always bound together and often exist on their own. Transient multi-protein complexes do also exist and are frequently found as part of cell signalling pathways, where protein interactions rapidly change in response to external signals and reversible post-translational modifications. These are generally referred to as dynamic molecular assemblies and are most challenging to study since they continuously change protein composition and can be very large and difficult to isolate from cells.

### Obligatory PPIs

**Figure 9:**
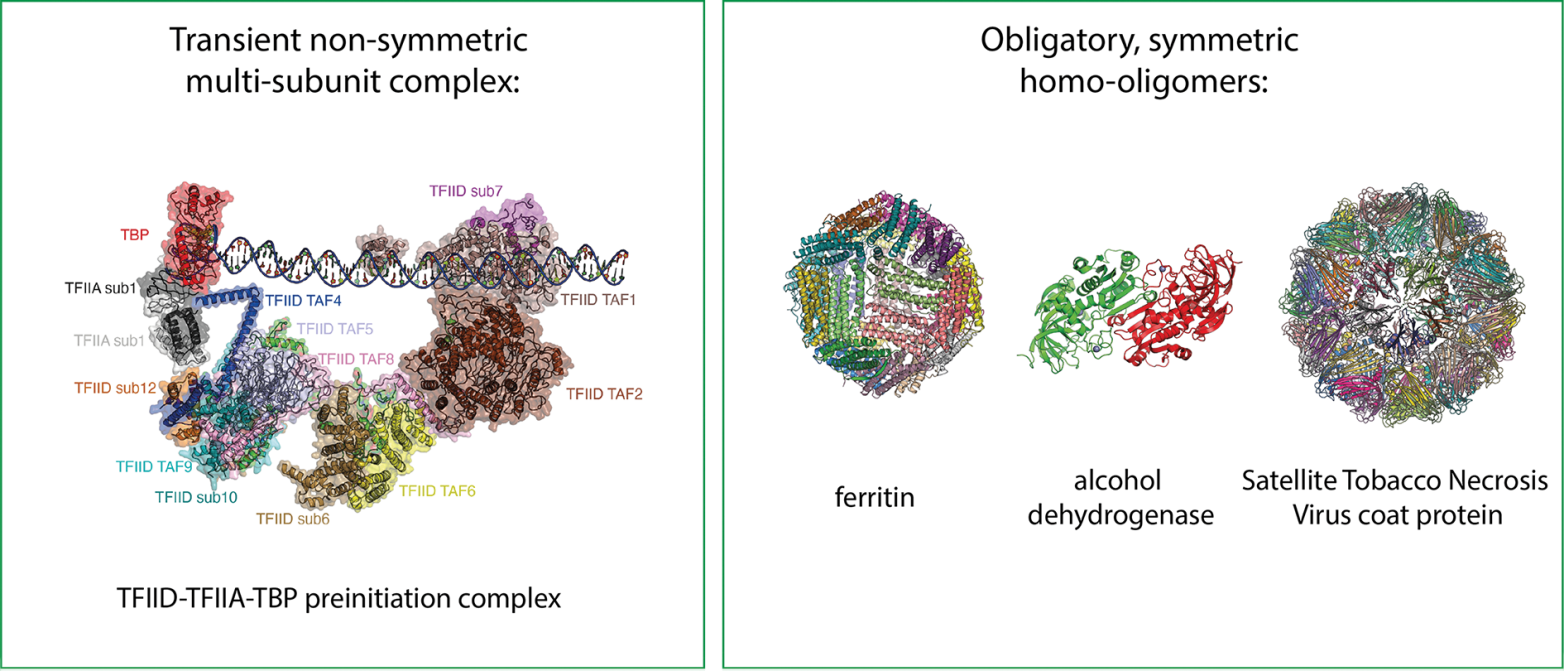
Examples of a non-symmetric transient multi-protein complex and some symmetric obligatory homooligomers The TFIID–TFIIA–TBP initiation complex is formed by many subunits that come together in a non-symmetric way to carry out a specific function. Ferritin, alcohol dehydrogenase and the Satellite Tobacco Necrosis Virus coat protein are examples of symmetric homooligomers, i.e. they comprise repeating subunits that form the functional unit. Each protein chain (subunit) in each example has a unique colour to highlight each component. PDB ID from left to right: 6MZM, 1HRS, 2OHX, 2BUK.

While reversible transient interactions are abundant, arguably, the most important protein interactions involve obligatory interactions. Proteins involved in these interactions do not exist on their own and instead self-associate to permanently exist as multiprotein assemblies or oligomers that define a proteins quaternary structure. Over 75% of *Escherichia coli* proteins are oligomers (with potentially a greater proportion in eukaryotes) and the majority are symmetric homooligomers, where the same protein chain (also referred to as a subunit) self-associates to form larger structures. For example, two subunits associate together to form a dimer, four subunits associate to form a tetramer, or twelve subunits associate together to form a dodecamer etc. Oligomeric proteins stay folded and are more stable than monomeric proteins, which protects them from degradation and aggregation, that usually require the unfolded chain to be exposed. Forming oligomeric proteins in a step-by-step process allows them to bind their targets cooperatively, which is a mechanism for increasing binding specificity (see section below). Furthermore, the subunit interfaces between two existing subunits evolve faster than the evolution of a new protein fold as subunit interfaces can easily form flexible active sites for enzymes or allosteric sites to regulate the overall structure and activity of the oligomeric protein. Non-symmetric, obligatory protein complexes also exist such as ribosomes, proteosomes, nuclear pores, chaperones, nucleosomes, and multi-enzyme complexes such as pyruvate dehydrogenase all of which contain a defined number of different proteins in a stable complex. These can make complex machines that have a defined function in the cell. Examples of both obligatory and transient protein interactions can be found in Figure 9.

**Figure 10:**
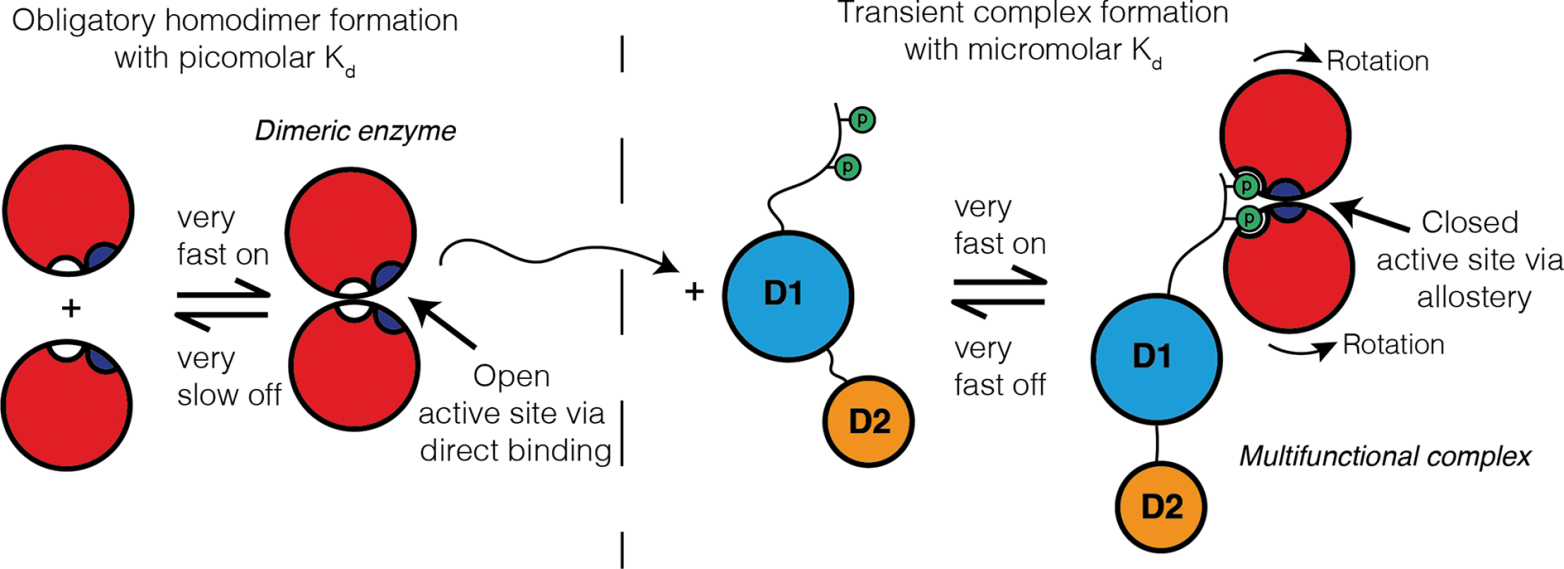
Two types of protein interaction On the left is an example of an obligatory homodimer between two folded monomers that creates an active site (coloured purple) between the subunits. On the right is an example of a transient multifunctional complex between the homodimer and an unstructured region containing two phosphorylated residues (indicated by the letter P) within a second multi-domain protein. The formation of the multifunctional complex causes the two monomers within the dimer to rotate to reveal secondary binding sites (white) and subsequently close the original active site (purple). This is an example of allostery, where binding of one protein changes the conformation of another, which may be controlled according to the presence/absence of post-translational modifications. The thermodynamic and kinetic parameters are different for the two types of interactions as indicated. The obligatory interaction may occur in a different compartment and subsequently move to another compartment (as indicated by the dashed line) to subsequently engage in the transient interaction.

A summary of the different types of protein interactions, surfaces, thermodynamics, and kinetics is illustrated in Figure 10. Now, we have defined the basic properties of PPIs and consider how these different parameters influence protein function inside the cell.

### Proteins need to find their targets quickly inside the cell

Inside the cell, many binding partners have high concentrations, which helps them find their targets quickly since the association rates is dependent on protein concentration. However, one factor that puts an upper limit to the association rate is the physical rate of diffusion that dictates the speed at which protein and target molecules move and collide with each other inside the aqueous environment of the cell. Another factor is whether there are available mechanisms for the protein and target to approach each other in the correct orientation so that the target sticks and does not simply bounce off and so has to try and collide with the protein again. This can be somewhat tricky for enzymes that have small active sites that are within a narrow crevice. Therefore, to increase the chance of a collision, the binding site may need a wider opening to be more exposed to allow targets to bind from multiple directions and orientations. Interestingly for some enzymes such as glucokinase, the enzyme partially populates an open conformation with a more exposed active site allowing functional catalytic rates of glucose conversion to glucose-6-phosphate.

**Figure 11:**
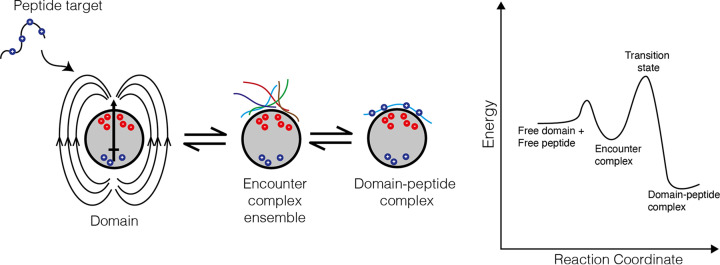
Electrostatics and the encounter complex in protein interactions A protein domain (grey) may have positive and negatively charged regions that generates electric force field lines that guides/orientates the positively charged peptide onto the negatively charged binding site. This allows it to dock in multiple ways on the surface of the protein via long-range and looser interactions in an encounter complex that facilitates a fast on-rate for the interaction (each peptide position is shown in a different colour). In the second step, as the correctly oriented peptide is experienced, it goes on to bind as the final complex. The encounter complex is a stable intermediate as shown in the energy diagram that describes a possible pathway from free peptide/domain to encounter to the final domain-peptide complex. See the uncovering protein structure review in this series for more information of these type of energy diagrams.

Proteins can also use long-range electrostatic interactions and a binding intermediate called the encounter complex to search the cell more efficiently for its specific partner. When a protein with net charge approaches close to its target that has an opposite charge, long-range attractive electrostatic interactions help it steer to the binding surface (Figure 11). Electrostatic effects are very common for protein transcription factors that bind DNA as the phosphate-sugar backbone is negatively charged. As a result of this, most of the transcription factors are positively charged to attract the DNA. Interestingly, many proteins inside the cell have the same charge, which keeps them from sticking to each other despite the relatively high concentrations of proteins found in cells. The formation of an intermediate binding state called the encounter complex further helps proteins bind their targets at functional faster rates. Intermediate states in biological reactions are useful as they enable the reaction to be separated into more easily attainable steps. This stepping through conformations rather than jumping to the end point has the effect of speeding up the overall reaction. In the encounter complex, the target initially binds to the protein in a different/looser way to its final arrangement/orientation. Crucially, however, this initial contact lasts long enough and is stable enough for the target to rearrange subtly on the surface to form the correct final complex and speed up the overall binding reaction (Figure 11).

**Figure 12:**
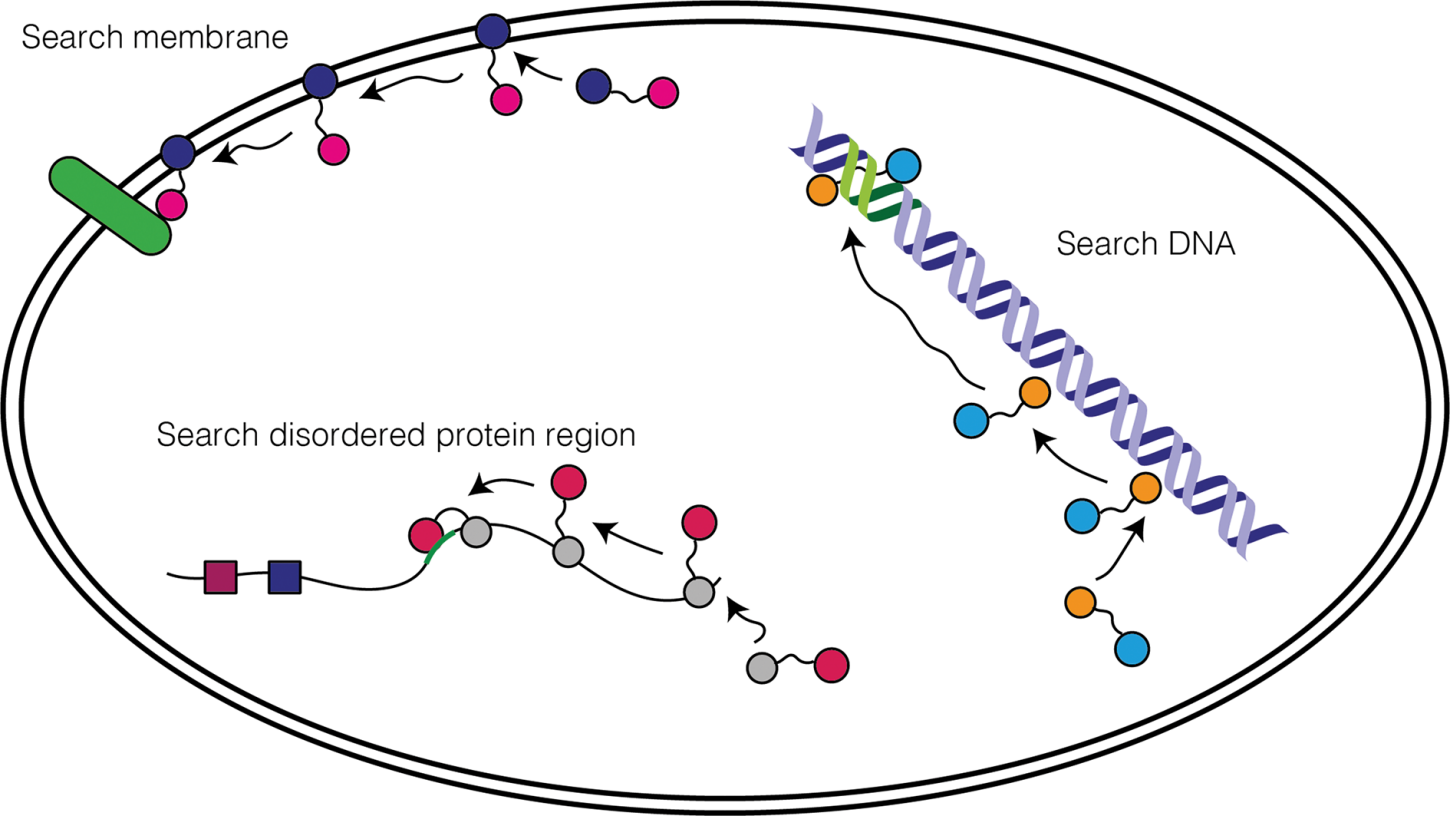
Proteins can find their partners faster by searching in 1D or 2D A multi-domain protein may first associate non-specifically with the DNA predominantly through one domain facilitated by long-range electrostatics to the charged DNA backbone and then ‘slide’ along DNA until its target DNA site (green) is found where the second domain can now also bind, locking it in place. Another multi-domain protein may bind a disordered region of a protein that is enriched in charged and hydrophobic residues which would allow non-specific binding and then slide or hop along the disordered region until its binding site (green) is found where again the second domain can now also bind, locking it in place. Another multi-domain protein may bind a lipid bilayer and diffuse until the second domain binds to a target membrane protein (green). In all cases, the search would be quicker since the protein is only scanning possible areas of the cell that are more likely to lead to the correct target. This is facilitated by having a separate domain that specialises in binding either DNA, disordered protein regions or lipid bilayers possibly through forming an encounter complex. In some cases, a single domain could achieve the same effect by having two distinct regions, a non-specific and specific binding surface.

Taken together, long-range electrostatic interactions and encounter complexes are both useful mechanisms to allow some proteins to find their partners faster. In many cases it will allow proteins to reduce their search from searching in three dimensions to two dimensions (i.e. searching within a charged membrane plane) or even one dimension (searching along a charged disordered protein region or DNA strand). In these cases, an initial (electrostatically driven) contact can be made rapidly with the search location (membrane, DNA strand, and disordered protein region) to form an encounter complex. Then, the protein can diffuse within the search location, which overall will be a smaller region to search as opposed to the whole 3D cell (Figure 12).

### Proteins need to bind their targets with specificity inside the cell

We have explored both thermodynamics and kinetics of protein interactions and how both favourable binding affinity and kinetics can be attained. However, the concept called macromolecular crowding brought about by the very high concentration of molecules inside the cell should also be considered. Macromolecular crowding means a protein will physically be in a smaller volume since the other molecules take up so much space and this alters its properties. For example, it will increase how much an individual protein associates with other proteins (target and non-target) but actually reduces the rate of association with its specific target (particularly for larger proteins). An analogy would be if you were trying to find a friend at a crowded party. If you simply randomly walk around, before you find your friend, you will bump into many other people. As such we must take into account how well proteins distinguish between targets and non-targets, i.e. the specificity of these interactions to understand the situation inside the cell.

**Figure 13:**
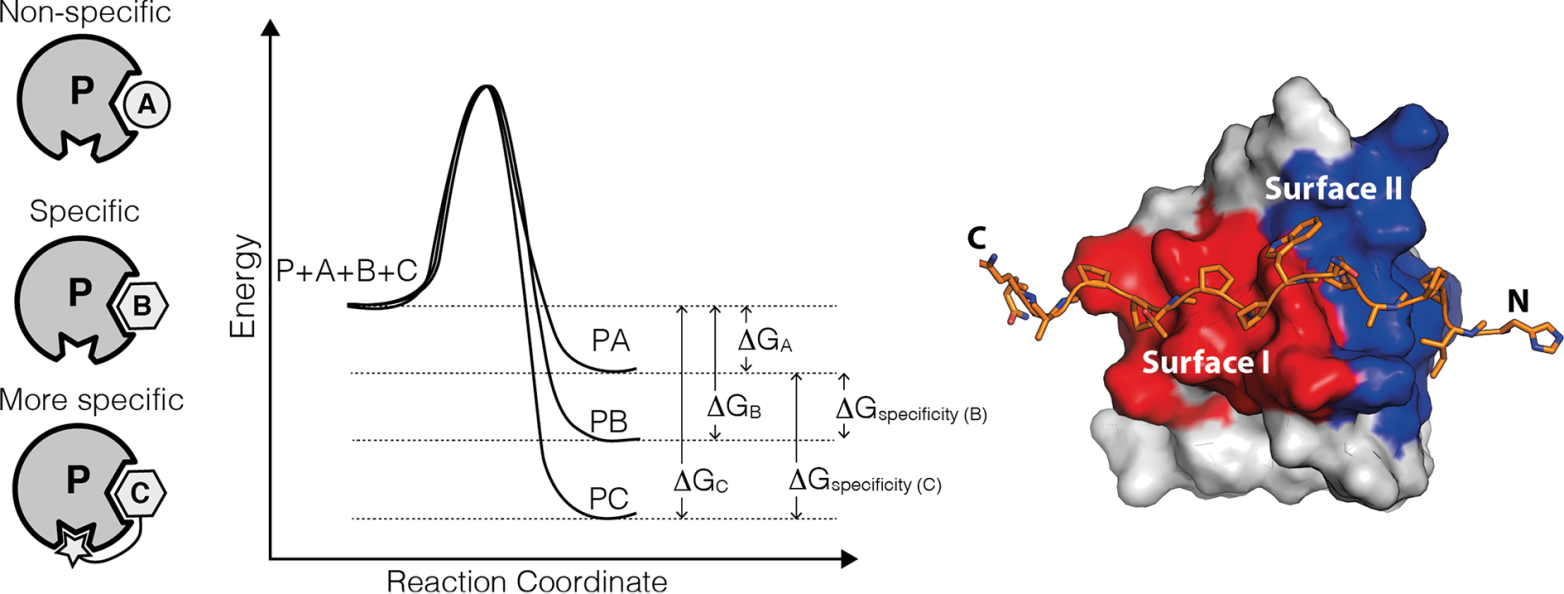
Free energy of specificity The protein P can bind a non-target A and two different targets B and C. The free energy of the protein complexes PA, PB, and PC are lower than the free energy of the components before binding and thus indicate favourable binding in all cases. However, the binding of B and C is more favourable than the binding of A, thus the difference in the binding free energy changes also known as the free energy of ‘specificity’ indicates how much better C and B binds than A. The bigger the difference the more specific the target is for the protein. C is more specific than B due to additional unique contacts with the protein. On the right is an example of an SH3 domain bound to an extended peptide that uses the common surface I (red) as well as an additional surface II (blue), which gives the interaction greater affinity and specificity (PDB ID: 2KXC).

As discussed above, protein binding is governed by the dissociation constant (*K*_d_) and the related Δ*G*^o^ or free energy of binding, however given that non-specific targets may also bind to a given protein, we must also define the free energy of ‘specificity’ for the interaction. The ‘specificity’ of the interaction is a relative thermodynamic term that tells us how well a given protein binds to its target compared with other potential targets in the cell (Figure 13). In other words, the specificity results from the relative affinities of the competing interactions. Binding becomes specific when the charge in free energy is larger for the target (i.e., peptide B and C) than it is for the non-target molecule (peptide A).

Besides increasing the interaction surface area as seen in complex P:C in Figure 13, another mechanism for increasing the free energy of specificity is by increasing the number of complete binding sites on the two interaction partners. For example, if a protein contains two protein interaction domains and its target contains two or more peptide target motifs, then it is less likely that an initial domain–peptide complex will dissociate as there are now more opportunities to bind via additional domain–peptide interactions. The cooperation between the two interaction domains to recognise tandem peptide targets that leads to higher affinity (and specificity) is known as the avidity effect and also is key to allowing improved binding kinetics as we saw in Figure 13.

### Summary

The most common way proteins exert their function is by binding to other molecules which is governed by the laws of thermodynamics and kinetics. Proteins interact with a range of binding affinities and these interactions are either transitory or obligatory, allowing dynamic molecular assemblies as well as stable higher order functional oligomers. Functional interactions often need to be rapid and specific to avoid many competing interactions inside the crowded cellular environment. As such, evolution has frequently employed long-range electrostatics and encounter complexes to help proteins find their biological targets quickly and increased interaction surfaces to ensure the interactions are specific. It is quite incredible that despite the high concentration of thousands of proteins, specific and sophisticated protein interactions are achieved resulting in a huge range of protein functions that direct life.

## Changes to protein function

The cellular environment is in constant flux, and proteins are required to rapidly respond to changes in the cellular environment. For this reason, cells have evolved various ways to modify or influence protein function. We have already considered several examples of these modifications throughout this review, especially considering PPIs. This section will focus in detail on the additional molecular mechanisms that can alter, influence and/or regulate protein function in cells including splicing and post-translational modifications.

The nature and effects of mutations on protein function will be introduced, before elaborating on how mutations allow the function of proteins to change over time due to evolutionary pressure. This section will conclude with a discussion of how small molecules and protein therapeutics can be designed to counteract the effect of mutations in disease.

### Splicing

**Figure 14:**
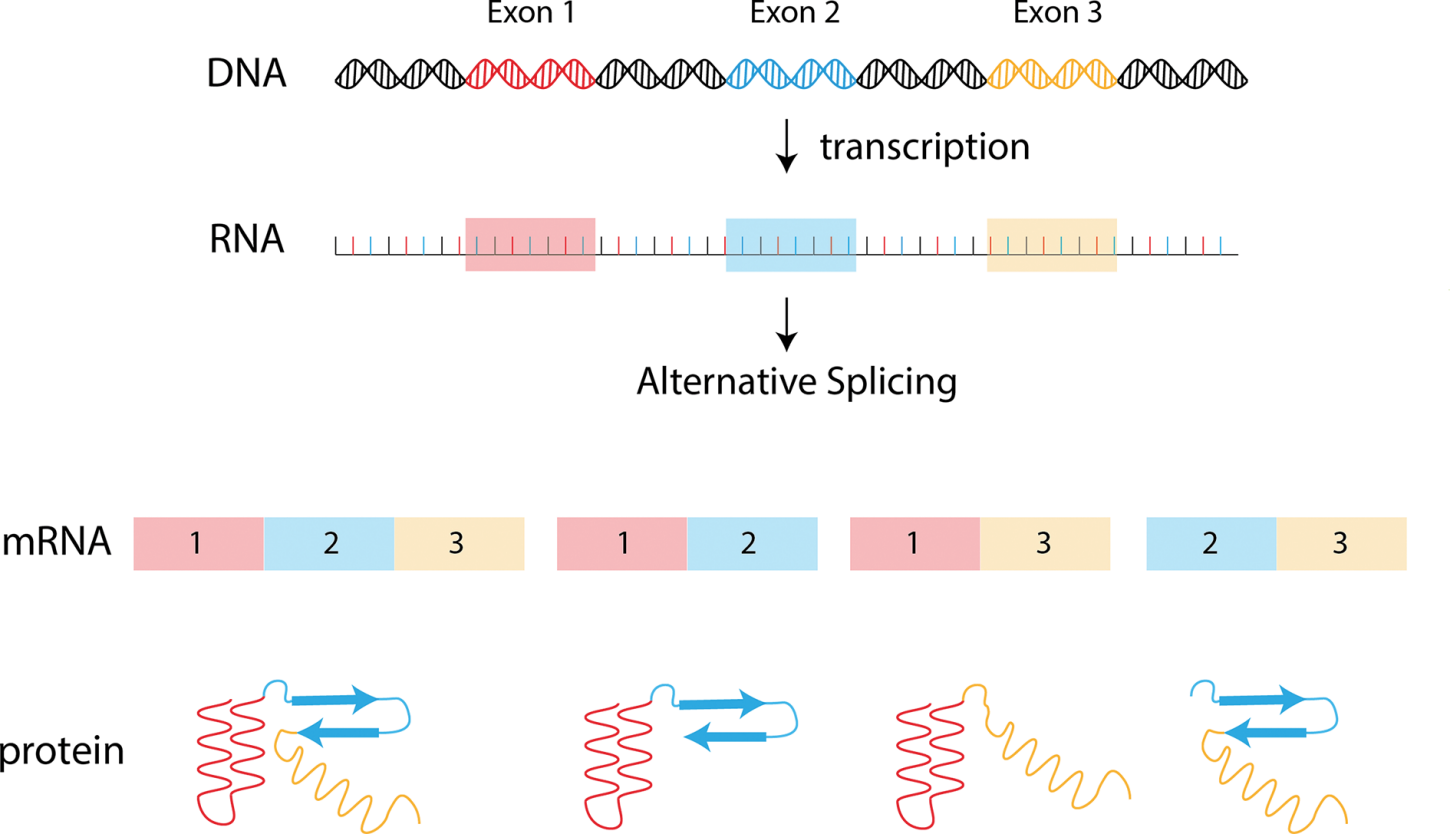
Mechanism of alternative splicing Genes consist of non-coding (introns) and coding (exons) regions. Each exon in this figure is indicated by a colour (blue, red, yellow) in this figure. Different arrangements of exons are ‘spliced’ together to make varying mRNA transcripts that give rise to proteins with different structural elements that may perform different functions.

Eukaryotic genes contain sequences termed introns and exons. Introns are generally considered non-coding regions of genes and get ‘spliced’ out of the final gene product. This leaves the exons that come together to encode specific proteins. The term that describes this process is gene splicing. Alternative splicing of RNA sequences can allow a single gene to generate more than one functional protein (Figure 14). These different functional proteins are termed isoforms, and it is estimated that in humans, approximately 30–65% of genes undergo alternative splicing to some degree. An example of a gene that undergoes alternative splicing resulting in more than one functionally distinct protein is MARCH3. One isoform of MARCH3 can down-regulate IL6Ra (a receptor for the cytokine IL-6) levels on the cell surface as it contains a putative PDZ-domain at the C-terminus, whereas another MARCH3 isoform does not contain this domain and is therefore unable to regulate levels of IL6Ra at the cell surface.

### Post translational modification

**Figure 15:**
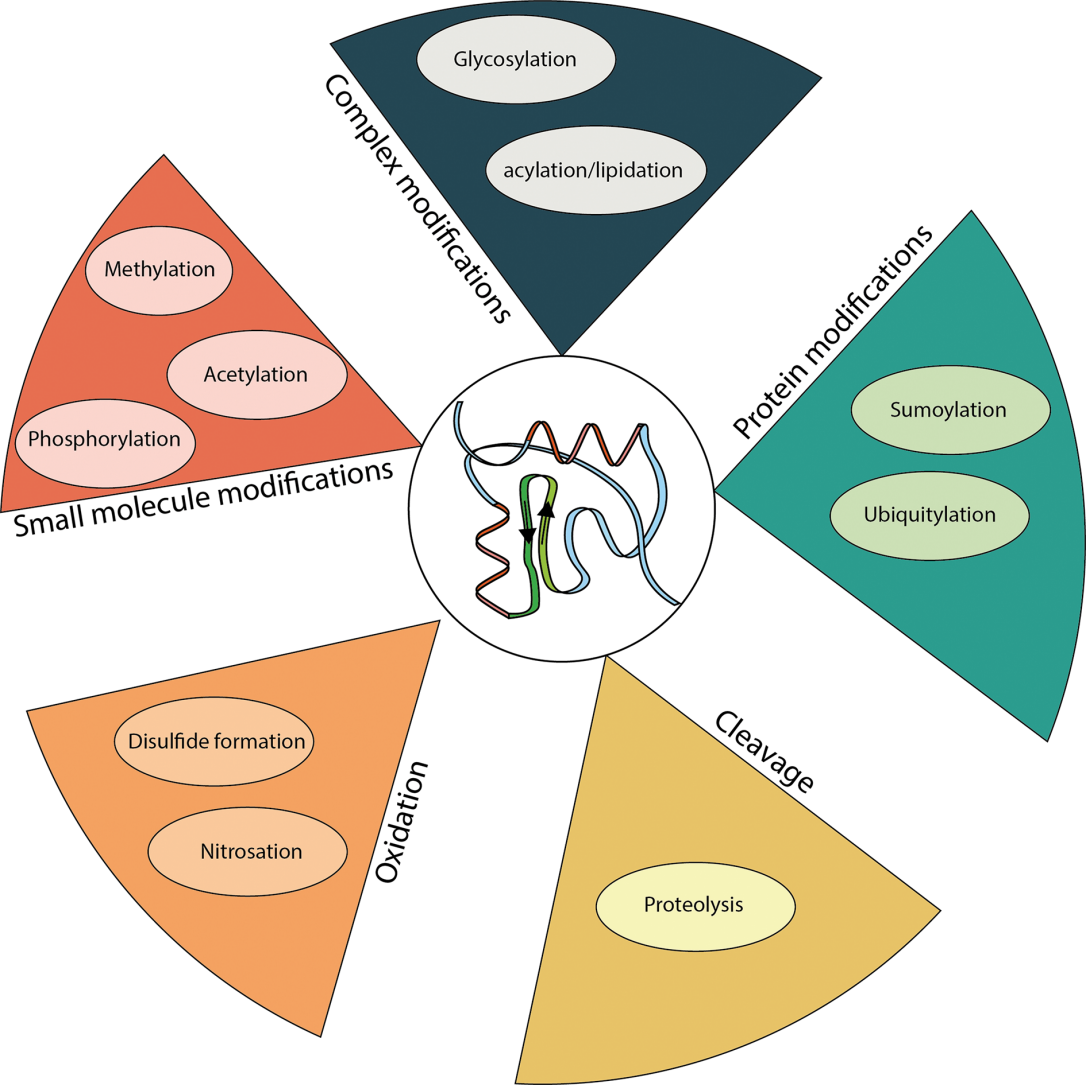
Overview of the various types of post translational modifications The different types of post-translational modifications can be classified depending on the modification type. Small molecule modifications include addition of small moieties such as methyl, phosphor, or acetyl groups to proteins. More complex modifications include glycosylation and lipidation. Other proteins such as SUMO and ubiquitin can be added to proteins. Proteolysis is a form of non-reversible cleavage, and finally disulphide formation and nitrosation involve oxidation of proteins.

Post-translational modifications (PTMs) are reversible or irreversible modifications made to proteins that occurs after their translation (Figure 15). PTMs are important for controlling protein function and increasing the functional diversity of specific proteins including inducing changes in enzyme activity, interaction with other molecules, cellular localisation, and stability. Estimates are that 5% of the whole proteome consists of enzymes which catalyse post-translational modifications including phosphatases, ligases, kinases, and transferases. Below we discuss select examples of some PTMs with a focus on how they can alter protein function and/or increase functional diversity.

### Phosphorylation

Protein phosphorylation is the addition of a negatively charged phosphate group to specific residues on proteins. It has been estimated that approximately 30% of eukaryotic proteins can be phosphorylated on at least one residue, making protein phosphorylation a common form of PTM. Phosphorylation predominately occurs on serine, threonine or tyrosine residues, with approximately 85% of phosphorylation in eukaryotes occurring on serine residues, and 12% and 2% on threonine and tyrosine residues, respectively. Phosphorylation events often serve to activate or inactivate protein activity, particularly in signal transduction where it is the most common form of PTM. The addition of the phosphate groups onto these residues changes the protein's surface charge, which in turn alters interactions with other proteins as well as interactions between residues within the protein itself. Kinases are enzymes that catalyse phosphorylation and phosphatases catalyse dephosphorylation. It is the balance between kinase and phosphatase activity that controls the constantly fluctuating phosphorylation level in the cell. Phosphorylation events are critical in cell cycle control, growth, signal transduction, apoptosis, and many more biological events.

### Ubiquitination

Ubiquitination involves the addition of a small protein called ubiquitin on to other proteins. This process involves a large family of proteins, the E2 and E3 ligases, that add ubiquitin molecules on to proteins, adaptor proteins that regulate ubiquitination, and deubiquitinating enzymes (DUBs) that reverse this process, removing ubiquitin chains. Ubiquitination of proteins is involved in the regulation of their stability, activity, localisation, and PPIs. Proteins can be mono- or poly-ubiquitinated, and different types of ubiquitin chains determine the effect ubiquitination has on the protein. Polyubiquitin chains consist of ubiquitin monomers that join via peptide or isopeptide bonds to form chains. Ubiquitination is most commonly associated with degradation, where the ubiquitin targets the protein either to the proteasome for degradation. However, there are also a number of other functional outcomes associated with ubiquitination. Table 4 provides a list of the different types of ubiquitin chain linkage types and the process this linkage type is associated with.

**Table 4:** Types of ubiquitin linkages and their associated function

Linkage type	Function
**K48**	Protein degradation
**K63**	Gene activation, DNA damage response, and scaffolding
**M1**	Gene activation, innate immunity, and scaffolding
**K6**	Mitophagy and DNA damage response
**K11**	Mitophagy, protein turnover trafficking, and degradation
**K27**	Autoimmunity, mitochondrial damage response, scaffolding, and degradation
**K29**	Degradation
**K33**	Trafficking and adaptive immunity
**K36-M1 hybrid**	Scaffolding

### Acetylation

Protein acetylation is one of the most abundant forms of PTM. Acetylation involves the transfer of acetyl groups from acetyl-CoA on to specific sites on polypeptide chains. Acetylation can occur on thiol groups (sulfur), hydroxyl groups (oxygen) and amino groups (nitrogen). Histone acetyl transferases and histone deacetylase enzymes play an essential role in the regulation of chromatin structure. Histone acetyl transferases are generally considered transcriptional coactivators as acetylation of lysine residues on histones is associated with relaxation of chromatin structure. Conversely, histone deacetylases, which remove acetyl groups from lysine residues, cause chromatin to compact and so are thought of as repressors. The balance of the actions of these proteins is essential in the regulation of gene expression. Histone acetyl transferases also target non-histone proteins including cytoskeletal proteins in the cytoplasm and transcription factors. Acetylation is also important for PPIs, metabolism, and protein localisation.

### Lipidation

Lipidation involves the addition of lipid moieties to proteins (Table 5). This makes proteins significantly more hydrophobic and can lead to increased interactions of the lipidated protein with biological membranes (as discussed in the protein binding section of this review). Lipidation can also influence PPIs, protein secretion, localisation, stability, and binding affinity. Lipid modification is tightly regulated by, and integrated with, cellular metabolism and signalling pathways. Lipid-based modifications of proteins are diverse in their nature and can be broadly grouped into six categories: fatty acids, isoprenoids, sterols, phospholipids, glycosylphosphatidylinositol (GPI) anchors, and lipid-derived electrophiles (LDEs). Lipids can modify a variety of amino acids, depending on their chemical nature, but generally, cysteines, serines, lysines or histidines may bear lipid modifications, in addition to the protein’s C-termini.

**Table 5:** Examples of types of lipid modification

Lipid modification	Function	Example of modification
**Fatty acylation**	Promotes weak protein–membrane and PPIs; key regulatory step in some signalling pathways	O-palmitoylation: Attachment of a palmitoleoylate group to a conserved serine on the signalling protein, Wnt, to enable binding to its receptor, the Frizzled G-protein coupled receptor
**Prenylation**	Mediates protein attachment to membranes	Linkage of an isoprenoid lipid on to a cysteine on the signalling protein Ras to localise it to the membrane
**Sterols**	Mediates attachment to plasma membrane and PPIs	Attachment of cholesterol to the C-terminal glycine of Hedgehog proteins, enables binding to its membrane bound receptor
**GPI-anchor**	Targets proteins to membranes	Attachment of GPI anchor to C-terminus of MSP-1, to localise it to the membrane and enable malaria parasite invasion
**Lipid-derived electrophiles (LDEs)**	Active lipid metabolites involved in signalling pathways	LDEs derived from prostaglandin J2 inhibit inflammation via binding to a critical cysteine of PPARγ

### Mutations

In addition to coordinated and controlled modification of protein function as discussed in the sections above, protein function can also change due to mutations, or changes in the sequence that codes for the protein. Unlike other forms of modification, mutations are uncontrolled and, in some cases, are detrimental to protein function. Mutations vary in their impact and may substantially alter protein function or have minimal or no detectable effect. The nature of the mutation and its position in the protein’s sequence both influence its effect on overall function.

For example, a mutation that occurs in the active site of an enzyme is more likely to have a severe effect on function than a mutation in a non-conserved region of the protein.

Mutations can occur spontaneously or be generated through errors in replication, during cell division or during DNA repair, or due to mutagenic damage to DNA. Mutations can be classified structurally, by their impact on the protein’s sequence, or by the functional impact on proteins. Structurally, mutations can occur at small-scale, affecting a small number of nucleotides through insertion, deletion, or substitutions of bases. Alternatively, mutations can involve large-scale modifications, through amplification, deletion, rearrangement, or fusion of long sections of DNA or chromosomes. BCR-ABL is an example of a fusion gene caused by reciprocal translocation of chromosomes 9 and 22. This fusion forms a constitutively active protein that induces uncontrolled cell division and the development of chronic myeloid leukemia.

Mutations within the coding regions of genes can change the resulting protein sequence. Such changes are classified into the four following categories:

- Missense mutations produce substitution of one amino acid for another. This may change the protein’s function, depending on the location of the mutation and whether the change is conservative (i.e., if the new amino acid has similar properties to the original residue.)
- Nonsense mutations occur when a change in amino acid results in the incorporation of a premature stop codon. This results in incomplete translation of the protein and will generally significantly disrupt its function. However, nonsense mutations near the C-terminal end of the protein sequence may enable normal function to be maintained.
- Silent mutations have no effect on the protein function as they involve no change in amino acid sequence, thus the overall structure of the protein remains the same despite the gene sequence itself being mutated.
- Frameshift mutations occur when there is a deletion or insertion of a number of bases not divisible by three. As codons are formed from triplets of bases, insertions and deletions alter the entire reading frame downstream, resulting in altered codons and so amino acid sequence. The size and location within the sequence determines the extent that the frameshift will affect protein function.

**Figure 16:**
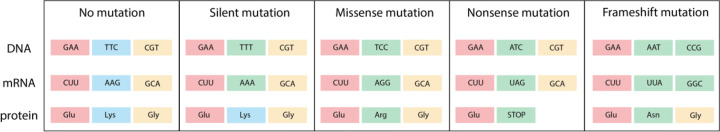
Four forms of mutation Silent mutations are due to a single base change that does not alter the final protein sequence whereas a missense mutation is a single base change resulting in a different amino acid being added to the protein sequence. A nonsense mutation leads to the addition of a premature stop codon within the protein. For ease of interpretation, green squares indicate the presence of a mutation in the DNA sequence, and where relevant, downstream changes to the mRNA or protein sequences based on the mutation.

The alterations caused by missense, nonsense, silent, and frameshift mutations are depicted in Figure 16.

The types of mutations discussed in the above sections are also classified according to their functional effect on proteins. Some mutations hamper the protein's function and are referred to as ‘loss of function’ mutations; likewise, mutations that enhance function are called ‘gain of function’. Using the Janus Kinase (JAK) proteins as an example we can examine the difference between a loss or gain of function mutation. The JAKs are a type of enzyme called a kinase and catalyse the addition of phosphate groups onto tyrosine residues in other signalling proteins. The C759R mutation in the JAK3 kinase (catalytic) domain results in inactivation of its kinase activity, leading to severe combined immunodeficiency (SCID) due to decreased immune signalling downstream of JAK3. Conversely, the V617F mutation in the regulatory domain of JAK2, generates an increase in kinase activity, resulting in over-activity and an association with blood cancers.

Some mutant genes can disrupt or override the function of wildtype proteins and so are ‘dominant-negative’. Nonsense mutations that induce the loss of a functional domain but retain a substrate binding domain exemplify this concept – the mutant will retain the ability to bind to its substrate but will block wild-type activity. This can occur, for example, if a transcription factor loses an activation domain, but retains its DNA binding domain. The mutant transcription factor will block the wildtype form from binding to its site (and thus is dominant) and will result in overall reduced transcription due to the absence of its activation domain (and so has a disruptive, or negative effect).

These are simplified examples of how mutations in proteins can activate or inactivate protein function. Table 6 presents examples of the different kinds of gain and loss of function mutations and some of the terms we use to describe these mutations.

**Table 6:** Types of gain of function and loss of function mutations

Kind of change	Terms used to describe
**Gain of function**	
*Increase in function*	Hypermorph, gain of function
*Interference with WT protein function*	Dominant negative, amorph
*Acquisition of new function*	Neomorph, dominant gain of function
**Loss of function**	
*Complete loss of function*	Amorph, null
*Reduction in function*	Hypomorph

### Evolution of protein function

Mutations are essential for driving the evolution of new protein functions. While the above section focused on mutations that can influence protein function during an organism's lifetime, mutations can also alter protein function over many generations through the process of evolution. Evolution allows the introduction of new protein functions into populations as well as adaptation of function to new niches or stressors. Many mutations have a harmful or deleterious effect on protein function, resulting in reduced host fitness. However, some mutations are beneficial, resulting in improved protein function or the development of a novel, useful function that enhances the fitness of the host organisms. For example, a mutation that produces a protein that engenders antibiotic resistance in a bacterium would improve the fitness of that bacterium, allowing it to survive, and pass on this novel protein function to its descendants. Some mutations are neutral, neither improving nor harming host fitness, but instead generating random variability that is known as ‘genetic drift’. Because there is strong selective pressure for active sites and functional motifs within proteins to be evolutionary conserved, diversity in protein function is often driven by gene duplication events. Duplication of a gene allows the function of the original gene to be maintained whilst the duplicate gene can be mutated, potentially generating novel protein forms, without harming the organism.

As a protein accumulates mutations over time, it becomes more different in sequence, structure, and function from its ancestor. Comparison of sequence and structure can therefore be used to define evolutionary and functional relationships between proteins. Proteins that share a common ancestor are often termed homologues, orthologues, or paralogues. Table 7 lists the definition of each of these.

**Table 7:** Definition of an orthologue, homologue, and paralogue

Category	Definition
Homologues	Proteins are considered homologous if they are derived from a common ancestor and can be either orthologues or paralogues of each other.
Orthologues	Orthologs are proteins (or domains) derived from speciation and are functionally equivalent genes in different organisms that usually have similar number/type of domains.
Paralogues	Paralogues are those proteins (or domains) which derive from duplicated genes within the same organism that have evolved new functions.

While most proteins have evolved towards one specific function, some proteins have also evolved additional functions. These proteins have been dubbed moonlighting proteins or are said to have moonlighting functions. This term comes from the term moonlighting used to describe having a second job in addition to your regular employment. See Table 8 for an example of moonlighting proteins from different kingdoms of life and examples of their functions.

**Table 8:** Examples of moonlighting proteins in different organisms

Kingdom	Organism	Protein	Main function and moonlighting function
**Animalia**	*Homo sapiens*	ERK2	MAP kinase and transcriptional repressor
**Plant**	*Arabidopsis thaliana*	Hexokinase 1	Metabolism and modulator of transcription
**Yeast**	*Saccharomyces cerevisiae*	Aconitase	Krebs cycle enzyme and mitochondrial DNA maintenance
**Prokaryote**	*Listeria monocytogenes*	GroEL	Protein chaperone and toxin
**Protist**	*Plasmodium vivax*	Aldolase	Glycolytic enzyme and host–cell invasion

Another example to illustrate the functional consequences of protein evolution is to consider extremophiles – organisms that can survive inhospitable environments such as extreme heat, cold, basicity, salinity, pressure, or radiation. Surviving in such conditions requires that proteins adapt to function in conditions that would otherwise denature them or otherwise severely limit activity. For example, high temperature typically causes irreversible unfolding of protein structure via exposure of the hydrophobic core of the protein. The heat-resistant proteins of thermophiles have evolved increases rigidity and stability, through forming higher-order oligomeric states or the introduction of stabilising salt bridges or disulfide bonds. Such mutations promote tighter protein packing and resistance to unfolding. This exemplifies how the accumulation of the simple mutations discussed in this section can lead to consequential changes to protein function, and so engender changes at an evolutionary scale.

### Small molecule drugs to modify protein function

Since proteins are so functionally important, it is not surprising that modulating their activity through drug binding is one of the most common ways drugs act as therapeutics. For example, a mutant protein that has increased mitogenic activity and so causes cancer (as discussed above) can be inhibited using a drug that changes the conformation of its active site or blocks binding to its substrate. One way of designing such a drug is through a process called structure-based drug design. This approach depends on analysing high-resolution structures of a protein to identify regions that can bind a small molecule inhibitor to have the desired effect. This has been a successful approach for developing drugs for HIV using HIV protease as a target. Drug companies are often interested in calculating the free energy of specificity which helps predict the likelihood of a drug having an unwanted side-effect by binding to other unintended proteins. One must also consider the kinetic profile of the protein:drug interaction (*K*_d_) as protein binding to target molecules is a dynamic process, with frequent association and dissociation events occurring. Thus the values for the drug association and dissociation rates are an important factor in drug development. As discussed earlier, since *K*_d_ is a ratio of these two values, a drug or any other target may bind a protein with the same affinity but can do so having fast association with fast dissociation or slow association with slow dissociation. In cell signalling, it is frequently desirable to have rapid association and dissociation at low ligand concentrations. However, for a drug, sometimes it is important for the drug to stay bound for a longer time so that frequent dosing is not required (Table 9).

**Table 9:** Summary of possible kinetic profiles for drugs

Profile	How does the compound behave	Pros	Cons
Fast on/Fast off	Rapidly reaches effective concentration at receptor, rapidly dissociates	Low dose required for effect hence good therapeutic index. Could be preferable if bioavailability is poor	Duration is determined by clearance rate of free drug hence frequent dosing required
Slow on / Slow off	Takes time to build to effective concentration at the receptor, slow dissociation once bound	Long duration allows once daily dosing, stable effects between peak and trough. Long duration even if the free drug is subject to rapid clearance	Slow on-rate, if plasma concentration is low, may mean that the drug never achieves sufficient receptor occupancy for efficacy

### Protein therapeutics

Proteins are much more versatile than small molecule drugs and may allow us to develop new therapeutics that can better modify the function of proteins associated with disease. For example, monoclonal antibodies specifically bind with high affinity and specificity to a protein target and can also be used to deliver a conjugated small-molecule drug to specific cells. However, monoclonal antibodies are expensive to produce, requiring animals to generate sensitised B-cells that excrete the specific antibody. Therapeutic proteins that can be produced directly in microorganisms such as yeast or bacteria offer much cheaper alternatives. In a relatively short time after the SARS-CoV-2 genome was published, small proteins were recombinantly produced to bind directly to the spike protein and inhibit viral entry. Many of these proteins don’t resemble any known protein and were either evolved in the lab or computationally designed against the known structure of the spike protein using artificial intelligence methods. These accomplishments indicate that directed evolution and these improved computational methods should allow us to manipulate many more protein functions with benefits in medicine, food production, the environment and many more areas in society.

### Summary

In this section, we looked at how protein function can be modified in various ways. Protein function can be altered before translation using alternative splicing mechanisms or after translation through reversible non-covalent modifications on individual residues. Protein function can also be altered by mutations which can be helpful or harmful to an organism depending on a number of factors. Mutations are also important for generating new protein functions through evolution. This process is oftentimes closely tied in with the niche the host organism inhabits, as clearly demonstrated by the unique environmental adaptations of extremophiles to their environment, that enhance their ability to survive, and thrive. Small molecule drugs and protein therapeutics are important tools to modulate protein function and treat disease, especially when mutations have occurred. The design of new functions and the modification of existing and emerging functions is closely linked to how complex multi-cellular organisms have evolved and develop. The better we understand how protein function is modified in living organisms, the closer we approach a more complete understanding of the molecular basis of life. We now look at a range of approaches to measure and determine protein function.

## Measuring and determining protein function

Our understanding of the function of proteins has been aided by our ability to not only solve protein structures but also measure protein function in the laboratory. Given the large breadth of techniques used to measure protein function it is not feasible to discuss them all. This section instead aims to provide some examples of the wide variety of functional assays and other techniques used to study proteins, with a particular focus on protein binding. For a more in depth look at other ways of characterising protein function we recommend reading the other reviews in this series which focus on specific topics such as ‘Enzymes: principles and biotechnological applications’ or ‘Uncovering protein structure’.

### Protein bioinformatics

Protein sequence alone can provide significant insights into aspects of protein function. Hence, computational analysis of sequence is a common foundational step prior to conducting experiments in the laboratory. There are several databases that summarise, annotate, and cross-reference information about proteins derived from the research literature. One such database is the Universal Protein Knowledgebase, also known as UniProt (uniport.org). UniProt is a comprehensive resource of key details related to proteins, including sequence, localisation, and function amongst many others. Other important computational approaches rely on comparing amino acid sequences between proteins. This allows the identification of closely related proteins or regions of conserved sequence, providing essential clues relating to function. Such analysis can also employ comparisons of protein folds or structures.

A significant breakthrough in computational biology was achieved in 2021 with the advent of AlphaFold, a new and powerful artificial intelligence tool. AlphaFold compares known protein sequences and structures to enable highly accurate predictions of most 3D protein structures from the amino acid sequence. Additionally, more recently it has enabled structural predictions of PPIs.

Structures provide unparalleled insights into how proteins work and accordingly there are several computation tools that enable deep analysis of structures. Perhaps most importantly, molecular graphics programmes such as Pymol allow complex structural data to be visualised and manipulated, allowing this information to be interpreted. Other programmes can identify cavities, channels, patches, or pockets that may form binding or catalytic sites, or depict charges and hydrophobicity distributions that may control binding kinetics. Structural bioinformatic analysis is powerful, however, not all proteins have a stable and defined 3D structure. Such proteins, known as ‘intrinsically disordered proteins’ are more difficult to interrogate with the tools described here. Bioinformatic tools have become impactful and indispensable in modern protein science, especially in the early stages of investigation. However, experimental approaches remain essential to validate computational predictions and to enable a full understanding of protein function.

### Measuring protein binding in the laboratory

Given that most proteins interact with other molecules in one way or another, it can be useful to investigate the nature, affinity, and functional consequences of protein interactions. Each technique has advantages and disadvantages and informs on different aspects of the interactions between protein and other molecules. Here, we will discuss the theory behind some techniques and how they allow us to probe protein interactions. While this does not represent the full repertoire of techniques that allow these kinds of interactions to be studied, we hope discussion of these techniques will provide some useful examples of some core approaches employed in the laboratory.

#### Phage display

**Figure 17:**
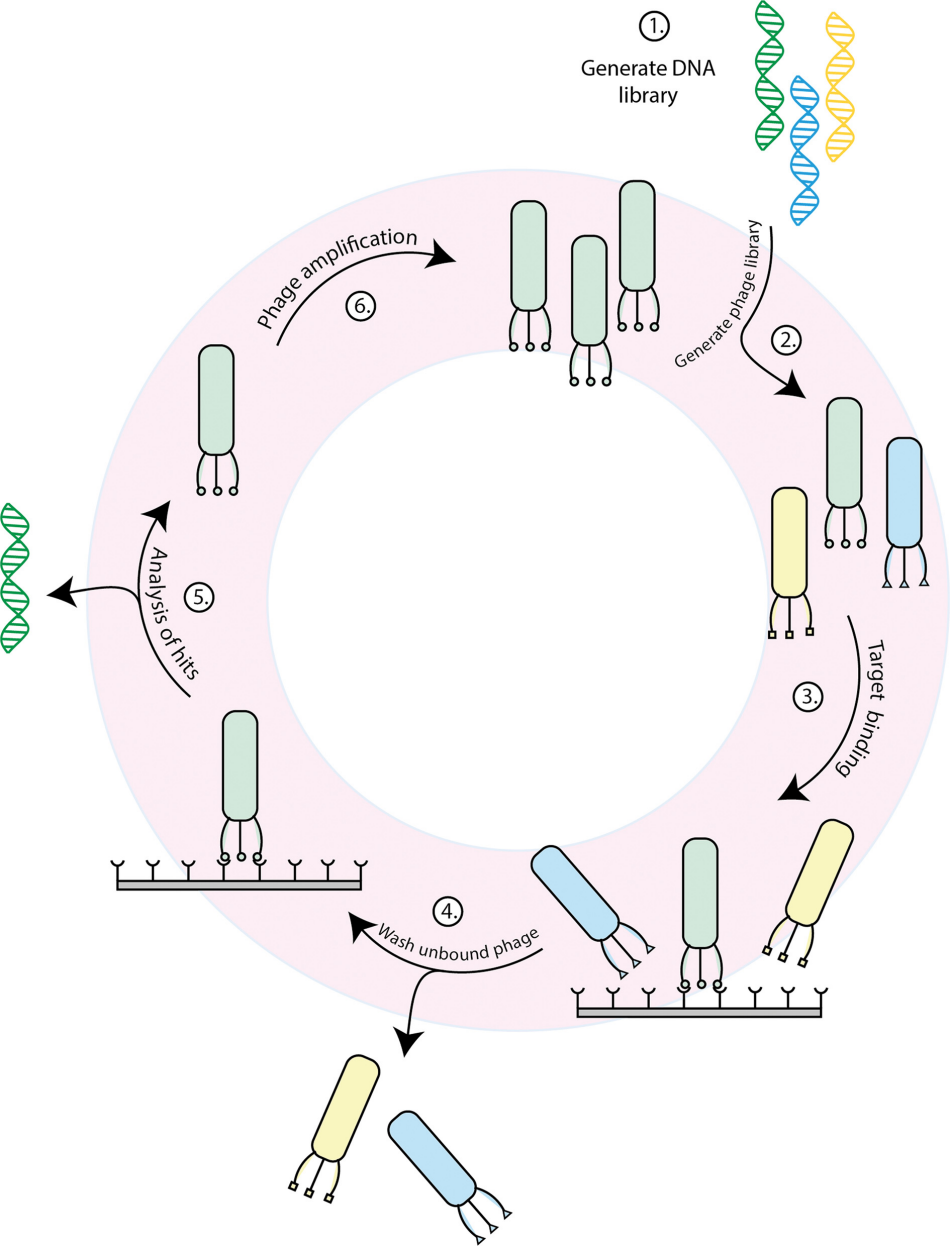
General process of biopanning with phage display libraries The first step involves generating and expressing a library of DNA that will encode the peptides or proteins to be displayed. This library will then be expressed in phage and tested for target binding. The target is immobilised and exposed to the phage library to allow binding to occur. During the binding step, the physical, chemical and biological parameters can be altered. Unbound phage are then washed away and the remaining bound phage are eluted. These phage can then be used to infect bacteria and generate more copies of the phage, from which the DNA sequence can be determined to identify the binding peptide or protein.

Phage display is a molecular biology technique for identifying molecules that interact with a protein. Phage display involves insertion of a gene encoding a peptide or protein of interest within the bacteriophage coat protein, causing the phage to display the protein on its surface. Large ‘phage display libraries’ can be made to rapidly screen many proteins against a target simultaneously. Binding between proteins in the library and a target is determined by a process termed biopanning. This involves immobilisation of the potential binding target, and subsequent incubation of this target with the phage display library. This is followed by cycles of washing, amplification, and reselection. The specific protein binding partner can be revealed by isolation and sequencing of the population of target-bound phage. This process results in the isolation of peptides, or proteins that are specific for a molecule of interest. This technique is depicted in Figure 17. While this technique is advantageous for screening large libraries of molecules for interactions, it is limited in that it can only measure binary interactions. An alternative version of this method, which is becoming increasingly popular is called yeast display, and involves fusing the protein of interest to a yeast cell wall agglutinin protein.

#### Fluorescence techniques

Fluorescence is a natural property that enables certain molecules to absorb light at specific wavelengths before subsequent emission of light at a longer wavelength. Fluorescent techniques are extensively used in molecular and cell biology, particularly in concert with spectroscopy and microscopy. Fluorescence indicators and labels allow molecules to be visualised or identified in cells and for a variety of kinetic and thermodynamic spectroscopic experiments to be carried out. Furthermore, more complex fluorescent properties, such as energy transfer, polarisation and fluorescence lifetimes are exploited in more advanced techniques. Here we will briefly discuss fluorescent proteins and a powerful fluorescent technique for investigating protein interactions, known as FRET.

##### Fluorescent fusion proteins

Green fluorescent protein (GFP) is a naturally fluorescent protein, that was first isolated from the jellyfish *Aequorea victoria* in the 1960s. Molecular biology methods allow GFP to be fused to proteins of interest, enabling fluorescence to be used as a gene expression reporter, biosensor or indicator of localisation using microscopy. GFP has been mutated to produce a variety of other fluorescent colours, such as red fluorescence protein (RFP) and yellow fluorescence protein (YFP) amongst many others. Fluorescent localisation experiments illustrate the power of GFP-fusions. The localisation of a protein within a cell can influence and inform upon its function, and this makes it useful for determining spatiotemporal information about a protein. For example, proteins in the nucleus typically have different functions to those found at the cell surface, and indeed proteins that translocate from one cellular compartment to another may exhibit different functions in different parts of the cell. By genetically engineering a GFP fusion to the N- or C- terminus of a protein of interest, it is straight forward to observe its position within a cell. Cells typically survive illumination of fluorescence proteins, facilitating live-cell imaging over periods of time. Several fluorescence fusions proteins of different colours can be used simultaneously to allow co-localisation experiments. Fluorescent fusion protein experiments require careful controls, as a potential caveat to their use is the modifications they can induce in their fusion partners. For example, many fluorescent proteins oligomerise and may induce unintended oligomerisation or aggregation of the target protein. Fluorescent proteins are also large, and this may affect the normal function or localisation of the fusion partner.

##### FRET

**Figure 18:**
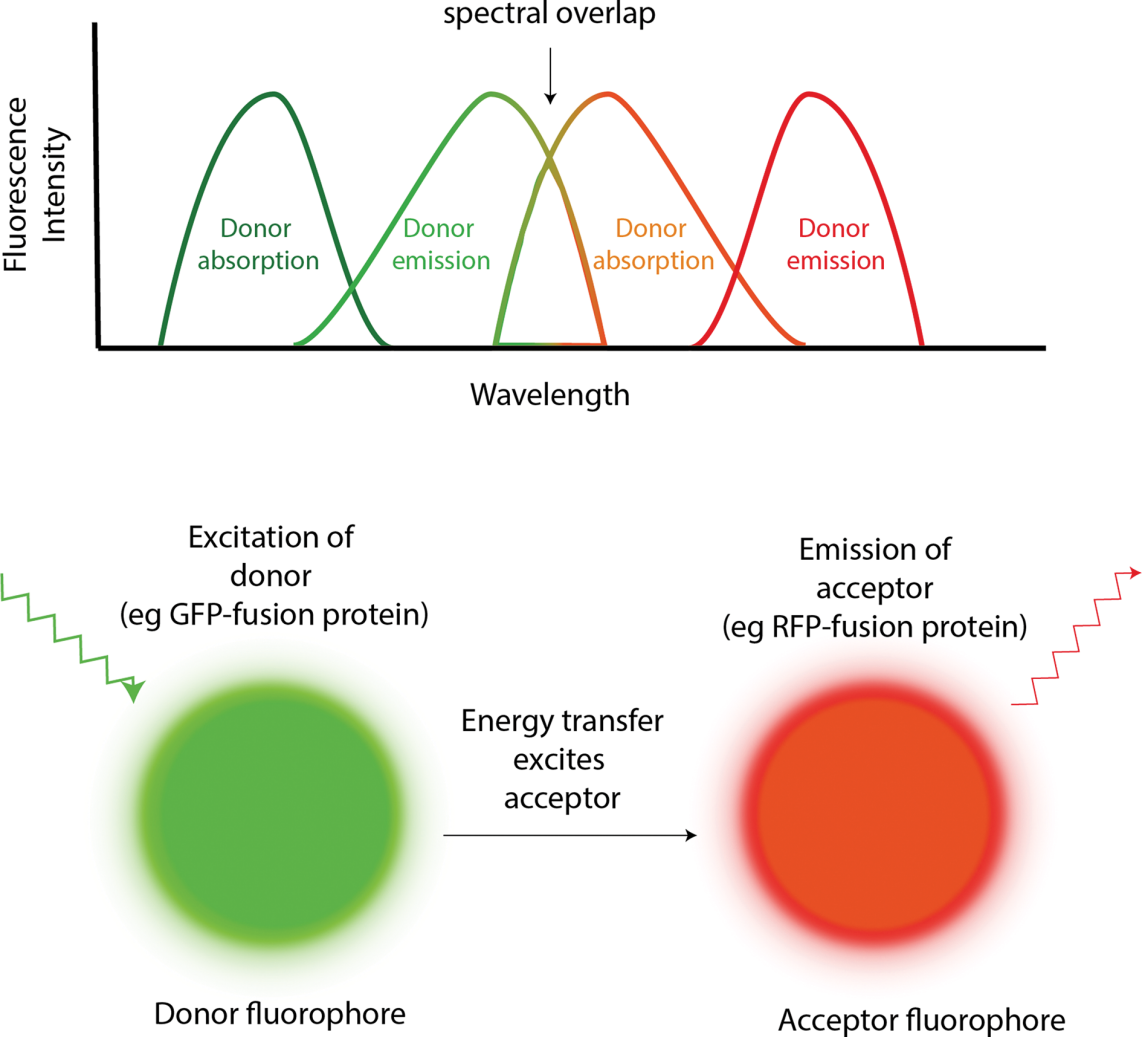
Overview of a typical FRET assay Two proteins of interest are labelled with fluorophores that form a FRET pair. One is a donor fluorophore (green) and the other is an acceptor (red). Each fluorophore has an absorption spectrum (shown on the left side in each colour) and an emission spectrum (shown on the right side in each colour). If the proteins are in close physical proximity, exciting the donor fluorophore will enable transfer of energy to the acceptor fluorophore, exciting it in turn, and enabling the interaction to be measured as emitted light from the acceptor fluorophore.

Fluorescent resonance energy transfer (FRET) is a fluorescence-based technique to measure interactions between two molecules. FRET involves two fluorophores that act as a pair. One fluorophore is known as the ‘donor’. When in an excited state, the donor transfers its excitation energy to its pair fluorophore, the ‘acceptor’. This energy is then measured as emitted light from the acceptor fluorophore (Figure 18). This transfer of energy only occurs if the two fluorophores have ‘spectral overlap’ – that is, the emission wavelength of the donor overlaps with the excitation wavelength of the acceptor, and if the two fluorophores are in close proximity, around 10 nm or less. By labelling two proteins of interest with fluorescent proteins such as green and red fluorescent proteins (a common FRET pair), it is possible to measure the interactions of such proteins, even in living cells using live cell imaging. Likewise, this approach can be used to show other dynamic cellular processes, such as the activation and transduction of signalling within cells. FRET is a powerful technique that can allow interactions to be observed that are difficult to interrogate by other methods, such as in live cells. However, results can also be complex to interpret, requiring careful controls and experimental design to measure what can be small changes in fluorescence.

#### Surface plasmon resonance

Surface plasmon resonance (SPR) provides information about affinity and binding kinetics between molecules. The basic idea of SPR is that binding of two molecules will change the refractive index which can be measured. In a simple SPR experiment, a protein, peptide, or small molecule is immobilised on a chip. Buffer is then flowed over the chip to determine the baseline refractive index. Following this, a second molecule (analyte) is flowed over the chip and if the two molecules interact, there will be a change in the refractive index, and from this the *k*_on_ can be determined. This part of the experiment is termed the association phase, and during this phase, a plateau will be reached when the association and dissociation of molecules are in equilibrium. Following the association phase, buffer is flowed over the chip again and the molecules will begin to dissociate, allowing *k*_off_ to be determined. The final step involves regeneration which will remove any bound molecules so the process can be repeated. The data generated by SPR is called a sensorgram, which provides information about association (*k*_on_) and dissociation (*k*_off_), which can be used to determine affinity of an interaction (*K*_d_). Sensorgrams will be measured at multiple analyte concentrations, and from the collective data, interaction kinetics can be accurately determined for *k*_on_, *k*_off_, and *K*_d_. While this technique provides a lot of useful information, it is limited in that you must be able to recombinantly produce your protein, and it must be stable and pure in order to accurately measure binding.

#### ITC

Isothermal titration calorimetry (ITC) is a technique that allows the thermodynamics of interactions between molecules in solution to be measured. In the context of proteins, ITC is useful for determining the binding constant, reaction stiochiometry, enthalpy and entropy of interactions of a protein with a ligand such as a peptide, another protein, or small molecule. ITC works by measuring heat released or absorbed when two molecules interact with one another using a microcalorimeter. This microcalorimeter contains two cells, one of which contains water and acts as a reference cell, and the other cell which contains the protein (the sample cell). A syringe is used to inject a series of small volumes of the ligand into the sample cell. If there is an interaction between the ligand and protein of interest the heat in the sample cell will change, and the machine will supply power to return this sample cell to the same temperature as the reference cell. As more ligand is injected into the sample cell, the protein will become saturated and so there will be less of a heat change. The resulting output from this is a series of peaks. The area under each peak is integrated and plotted against the molar ratio of ligand to protein. These data are then fitted to a binding curve which allows the affinity (*K*_d_), stoichiometry and binding enthalpy to be directly determined and the binding entropy determined indirectly. As with SPR, ITC also required recombinantly produced protein that is stable and pure.

#### Liposome assays

**Figure 19:**
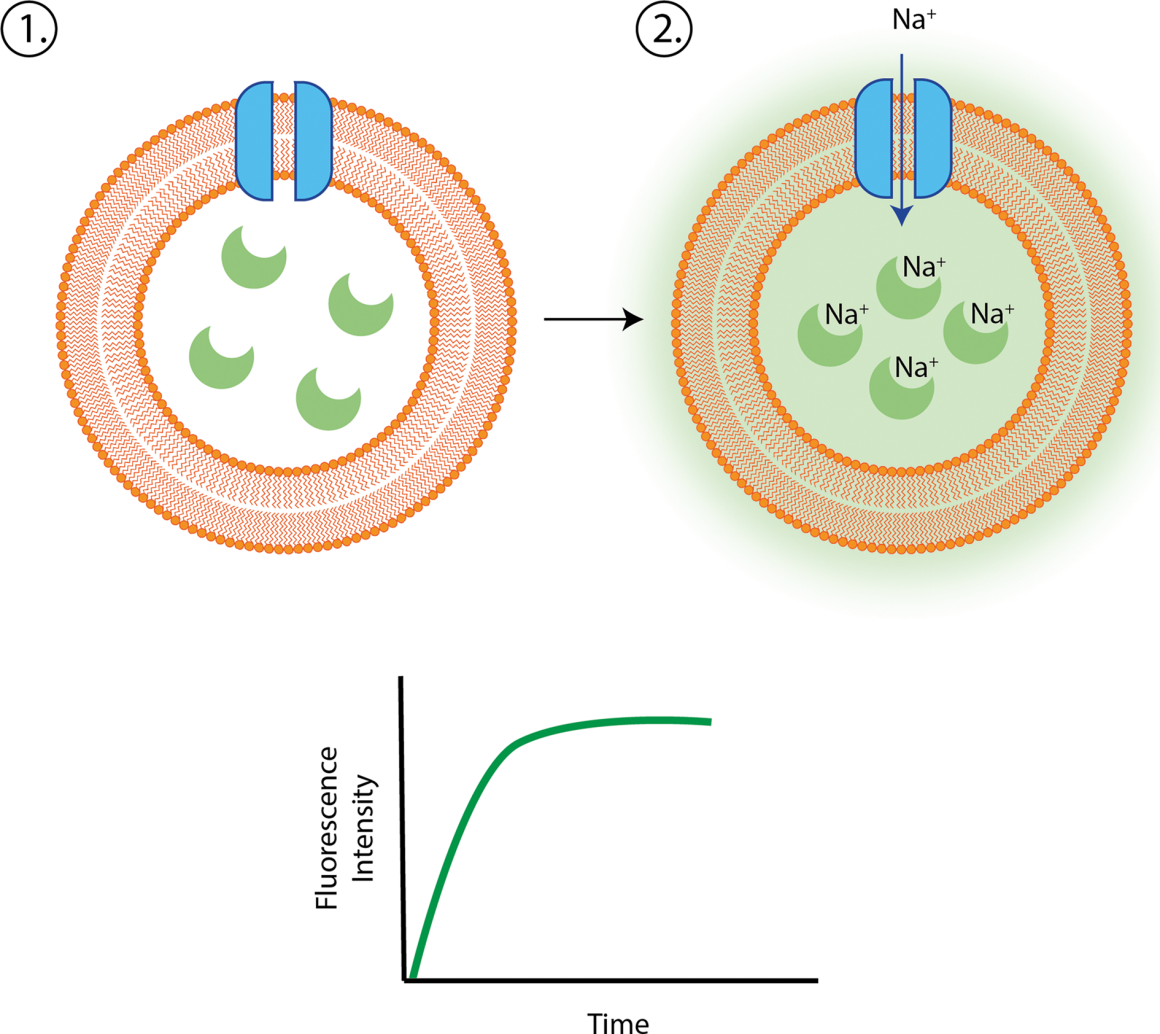
Overview of a liposome assay A liposome forms an artificial membrane environment that contains a sodium channel (blue) and encapsulates a sodium sensitive fluorescent dye (green crescents). Sodium ions are transported into the liposomal lumen by the channel whereby they interact with the fluorescent dye, resulting in an increased fluorescent readout (bottom).

Many proteins interact with or function across a membrane, and lipid vesicles, also known as liposomes, provide a means to artificially replicate this environment in an assay. Liposomes structurally resemble cell membranes, in that they form a single bilayer, with an aqueous environment on both sides of the membrane. Liposomes can be created in a range of sizes and the lipid composition can be modified to exactly match the arrangement of lipids in natural membranes. Liposome-based assays are used to measure the function of receptors, transporters, channels, pore-forming proteins, and lipid-binding proteins. Measuring protein–lipid binding typically takes the form of flotation assays, in which protein and liposomes are centrifuged in a sucrose gradient, allowing unbound proteins to separate from membrane-bound proteins. Channels and transporters are interrogated using liposome-based transport assays, typically involving the accumulation of a radiolabelled substrate or the linkage of substrate transport to a change in intensity of a fluorescent indicator. The function of the pore-forming proteins make use of ‘dye-release’ assays. A dye is held within the aqueous core of the liposome in close contact to a quencher, but after the formation of a pore, the dye will escape the quencher and the increased fluorescence can be measured in a continuous assay (Figure 19). Liposomes are one of the few ways to closely replicate biological membranes outside of the cell; however, they can be difficult to make and work with, and it can be challenging to incorporate well-folded membrane proteins into the membrane.

### Summary

**Figure 20:**
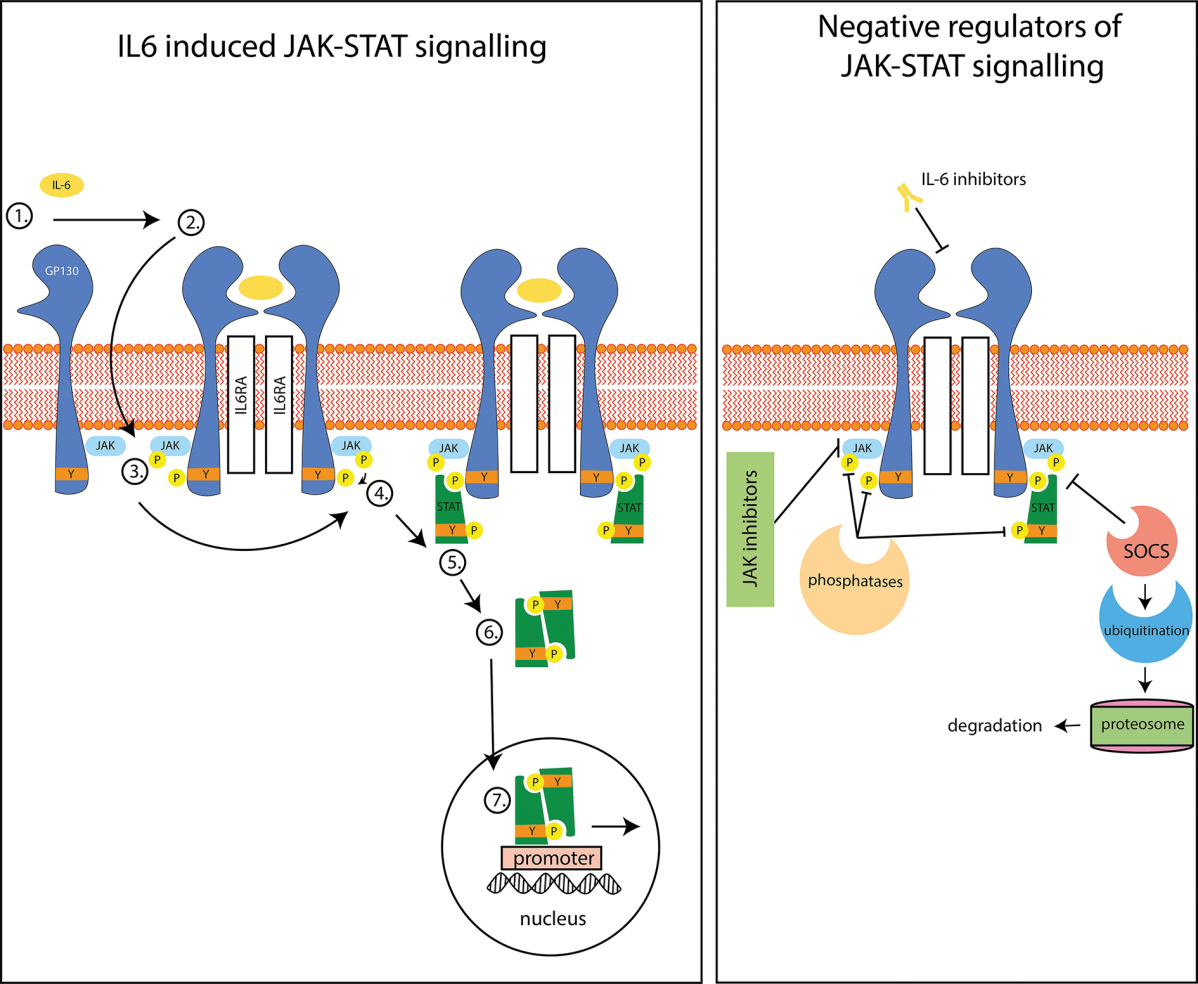
Schematic outlining the IL-6 JAK-STAT signalling pathway and regulation The JAK proteins are constitutively associated with the GP130 receptor. IL-6 binds IL6RA and GP130, allowing the JAKs to come into close proximality to one another. The JAKs are then activated by transphosphorylation, and subsequently phosphorylate nearby tyrosine residues including those found on the GP130 receptor tail. The four distal phosphotyrosine sites act as docking sites for STATs. Activated STATs then translocate to the nucleus where they bind to specific regions on target DNA and up-regulate gene transcription. These changes in gene transcription dictate the response the cell has to the initial cytokine binding. There are several proteins involved in the regulation of JAK-STAT signalling, including the SOCS proteins and phosphatases which control ubiquitination and dephosphorylation of the proteins, respectively. Additionally, drugs such as JAK and IL-6 inhibitors have been developed as therapeutics to block signalling via the JAK-STAT signalling pathway.

Measuring protein function in the laboratory has been fundamental to our ability to understand and influence protein function, particularly when it comes to thinking about how we can therapeutically target proteins or use protein function to treat disease. This section provided an overview of various techniques we employ in the laboratory to study and better understand protein function with a focus on protein interactions (Figure 20).

## Integration of key concepts – a biological example

To illustrate how many of the concepts presented in this review integrate with one another, below we present an example of a biological process with the key concepts discussed throughout this review bolded. This section should serve as an instance of how the functions of specific proteins and their modification come together to carry out a specific biological process. Here we use cytokine signalling as an example.

There are over 50 cytokines, and each is important for coordinating haematopoiesis, the immune response and inducing inflammation. Cytokines are small signalling proteins that bind specific receptors found on the surface of cells. An example of a cytokine is interleukin-6 (IL-6), which is released from various cells in the body and binds to the cell surface receptor, IL6ra. Upon binding to IL6Ra, the IL-6:IL6ra complex can then associate with another receptor, glycoprotein 130 (GP130). GP130 is a transmembrane protein, with an extracellular portion and an intracellular portion (IL6ra does not contain an intracellular portion). The extracellular portion forms the ternary complex with IL-6 and IL6Ra, while the intracellular portion is essential from downstream signalling inside the cell. Formation of the IL-6:IL6Ra:GP130 signalling complex at the cell surface leads to activation of the JAK proteins inside the cell as they come into close proximity. The JAK proteins are constitutively associated with the GP130 receptor tail and they activate one another by transphoshorylation of specific tyrosine residues within their kinase domain. Once activated, the JAK proteins can then phosphorylate other nearby proteins, including the GP130 receptor tail which contains several tyrosine residues that can be phosphorylated. These phosphotyrosine residues can then act as docking sites for proteins that contain SH2 domains such as the signal transducer and activator of transcription (STAT) proteins. Binding of the STATs to the receptor via their SH2 domain results in phosphorylation of the STAT at a C-terminal tyrosine residue. This phosphorylation event leads to a conformational change into their active form and allows the phosphorylated STATs to dimerise. Once in their activated dimer form, they translocate into the nucleus where they bind to regulatory regions on target DNA and up-regulate gene transcription. These changes in gene transcription lead to the eventual biological outcome whether that be differentiation, division, inflammation etc. One of the genes that is up-regulated encodes the suppressor of cytokine signalling (SOCS) proteins which are negative regulators of the JAK-STAT pathway. The SOCS proteins are multi domain proteins – they comprise a variable N-terminal region, a central SH2 domain and a c-terminal SOCS box. The SOCS proteins can directly inhibit the JAK protein activity by inserting an inhibitory region into the active site of the JAK via a region in the N-terminal (for SOCS1 and SOCS3). In the case of SOCS3, specificity for regulating specific cytokines comes from binding of the SH2 domain to the GP130 cytokine receptor. Alternatively, the SOCS proteins that do not contain this inhibitory region can recruit an E3 ligase complex via their SOCS box to the receptor/JAK. This E3 ligase domain leads to ubiquitination of the JAK-STAT proteins, tagging them for degradation. Other proteins that regulate the JAK-STAT pathway include phosphatases which remove the phosphate groups from activated proteins. It is important for these proteins to regulate signalling, as too much or too little signalling can lead to disease. Mutations in the proteins of the JAK-STAT pathway are therefore linked with several haematological, immunological and inflammatory diseases. As such, there have been drugs and therapies designed to target proteins involved in cytokine signalling to treat various diseases. For example, the JAK2 V617F mutation is the most common mutation in a group of diseases called the myeloproliferative neoplasms. These diseases are driven by increased and uncontrolled signalling downstream of JAK2, and so kinase inhibitors such as ruxolitinib bind to the active site of the JAK2 kinase domain and inhibit their activity.

## Concluding comments

We hope this review has helped with understanding of the following concepts:

- Functions of protein domains and the range of protein functions
- How evolution has shaped protein function
- The range of protein interactions and how they shape function
- How post-translational modifications can influence protein function
- How environment, oligomerisation, splicing, and mutations can lead to changes in protein function
- Some of the methods we use to measure protein function in the laboratory

## Open Access

Open access for this article was enabled by the participation of University Of Melbourne in an all-inclusive *Read & Publish* agreement with Portland Press and the Biochemical Society under a transformative agreement with CAUL.

## Abbreviations

3D: three-dimensional
CRISPR: Clustered Regularly Interspaced Short Palindromic Repeats
DNA: deoxyribonucleic acid
FRET: fluorescent resonance energy transfer
GP130: glycoprotein 130
HIV: human immunodeficiency virus
IL: interleukin
ITC: isothermal titration calorimetry
JAK: janus kinase
PAGE: polyacrylamide gel electrophoresis
PDB: protein database
PPI: protein–protein interaction
PTB: phosphotyrosine binding
PTM: post-translational modification
SAM: sterile α motif
SCID: severe combined immunodeficiency
SH2: Src homology 2
SOCS: suppressor of cytokine signalling
SPR: surface plasmon resonance
STAT: signal transducer and activator of transcription
TCA: tricarboxylic acid
TRAP: Trp RNA-binding attenuation protein
Trp: tryptophan
UBA: Ubiquitin association

## References

[B1] Watch a video on proteins and see their range of structures and functions: https://pdb101.rcsb.org/learn/videos/what-is-a-protein-video (Feb, 2022)

[B2] Explore protein folding on your computer with the game Foldit: https://fold.it/ (Feb, 2022)

[B3] Read about how *de novo* proteins promise new covid vaccines and medicines: https://www.scientificamerican.com/article/artificial-proteins-never-seen-in-the-natural-world-are-becoming-new-covid-vaccines-and-medicines/ (Feb, 2022)

[B4] Look at the predicted structures of proteins in the AlphaFold database: https://alphafold.ebi.ac.uk/ (Feb, 2022)

[B5] Learn more about biophysical techniques for measuring protein function: https://portlandpress.com/emergtoplifesci/article-abstract/5/1/1/228110/Evolution-of-biophysical-tools-for-quantitative (Feb, 2022)

